# Cross-prediction-powered inference

**DOI:** 10.1073/pnas.2322083121

**Published:** 2024-04-03

**Authors:** Tijana Zrnic, Emmanuel J. Candès

**Affiliations:** ^a^Department of Statistics, Stanford University, Stanford, CA 94305; ^b^Stanford Data Science, Stanford University, Stanford, CA 94305; ^c^Department of Mathematics, Stanford University, Stanford, CA 94305

**Keywords:** statistical inference, CIs, machine learning, prediction

## Abstract

While reliable data-driven decision-making hinges on high-quality labeled data, the acquisition of quality labels often involves laborious human annotations or slow and expensive scientific measurements. Machine learning is becoming an appealing alternative as sophisticated predictive techniques are being used to quickly and cheaply produce large amounts of predicted labels; e.g., predicted protein structures are used to supplement experimentally derived structures, predictions of socioeconomic indicators from satellite imagery are used to supplement accurate survey data, and so on. Since predictions are imperfect and potentially biased, this practice brings into question the validity of downstream inferences. We introduce cross-prediction: a method for valid inference powered by machine learning. With a small labeled dataset and a large unlabeled dataset, cross-prediction imputes the missing labels via machine learning and applies a form of debiasing to remedy the prediction inaccuracies. The resulting inferences achieve the desired error probability and are more powerful than those that only leverage the labeled data. Closely related is the recent proposal of prediction-powered inference [A. N. Angelopoulos, S. Bates, C. Fannjiang, M. I. Jordan, T. Zrnic, *Science* **382**, 669–674 (2023)], which assumes that a good pretrained model is already available. We show that cross-prediction is consistently more powerful than an adaptation of prediction-powered inference in which a fraction of the labeled data is split off and used to train the model. Finally, we observe that cross-prediction gives more stable conclusions than its competitors; its CIs typically have significantly lower variability.

## Significance

Machine learning is increasingly used as an efficient substitute for traditional data collection when the latter is challenging. For example, predictions of conditions such as poverty, deforestation, and population density based on satellite imagery are used to supplement accurate survey data, which requires significant time and resources to collect. However, predictions are imperfect and potentially biased, calling into question the validity of conclusions drawn from such data. This manuscript introduces a method for valid inference powered by machine learning. The method enables researchers to draw more reliable and accurate conclusions from machine learning predictions.

As data-driven decisions fuel progress across science and technology, ensuring that such decisions are reliable is of critical importance. The reliability of data-driven decision-making rests on having access to high-quality data on one hand, and properly accounting for uncertainty on the other.

One frequently discussed issue is that acquiring high-quality data often involves laborious human labeling, or slow and expensive scientific measurements, or overcoming privacy concerns when human subjects are involved. Machine learning offers a promising alternative: Sophisticated techniques such as generative modeling and deep neural networks are being used to cheaply produce large amounts of data that would otherwise be too expensive or time-consuming to collect. For example, tools to predict protein structure are supporting wide-ranging research in biology (1–4); large language models are being used to generate difficult-to-aggregate information about materials that can be used to fight climate change (5); predictions of socioeconomic and environmental conditions based on satellite imagery are being used for downstream policy decisions (6–9). This increasingly common practice, marked by supplementing high-quality data with machine learning outputs, calls for new principles of uncertainty quantification.

In this work, we study this problem in the semisupervised context, where labels are scarce but features are abundant. For example, precise measurements of environmental conditions are difficult to come by but satellite imagery is abundant. Due to its volume, satellite imagery is routinely used in combination with computer vision algorithms to predict a range of factors on a global scale, including deforestation (10), poverty rates (6), and population densities (11). These predictions provide a compelling substitute for resource-intensive ground-based measurements and surveys. However, it is crucial to acknowledge that, while promising, the predictions are not infallible. Consequently, downstream inferences that uncritically treat them as ground truth will be invalid.

We introduce cross-prediction: a broadly applicable method for semisupervised inference that leverages the power of machine learning while retaining validity. Assume a researcher holds both a small labeled dataset and a large unlabeled dataset, and they seek inference—i.e., a *P*-value or a CI—about a population-level quantity such as the mean outcome or a regression coefficient. Cross-prediction carefully leverages black-box machine learning to impute the missing labels, resulting in both valid and powerful inferences. The validity is a result of a particular debiasing step; the power is a result of using sophisticated predictive techniques such as deep learning or random forests. We show that the use of black-box predictions on the unlabeled data can lead to a massive improvement in statistical power compared to using the labeled data alone.

**Fig. 1.**
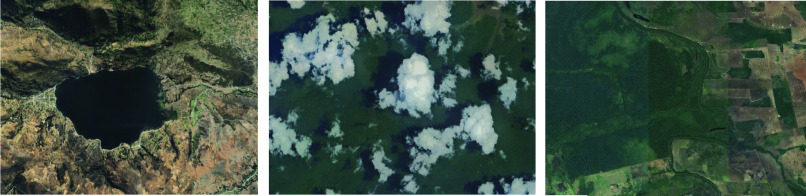
Examples of GEE satellite imagery used in the deforestation analysis of Bullock et al. (13).

**Fig. 2.**
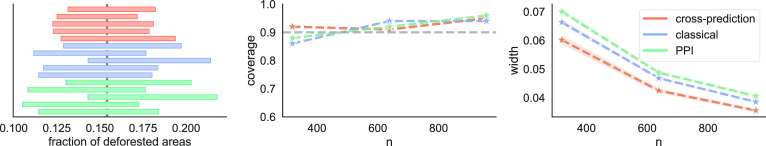
Estimating the deforestation rate in the Amazon from satellite imagery. *Left*: Example intervals constructed by cross-prediction, classical inference, and predictionpowered inference (PPI), for five random splits into labeled and unlabeled data and a fixed number of gold-standard deforestation labels, *n* = 319. *Middle and Right*: Coverage and interval width averaged over 100 random splits into labeled and unlabeled data, for *n ∈* {319, 638, 957}. The target of inference is the fraction of the Amazon rainforest lost between 2000 and 2015 (gray line in *Left* panel). The target coverage is 90% (gray line in *Middle panel*).

Cross-prediction builds upon the recent proposal of prediction-powered inference (12). Unlike prediction-powered inference, we do not assume that our researcher already has access to a predictive model for imputing the labels. Rather, to apply prediction-powered inference, the researcher would need to use a portion of the labeled data to either train a model from scratch or fine-tune an off-the-shelf model. We show that this leads to a suboptimal solution. Consider the following example studied by Angelopoulos et al. (12). The goal is to form a CI for the fraction of the Amazon rainforest that was lost between 2000 and 2015. A small number of “gold-standard” deforestation labels for certain parcels of land are available, having been collected through field visits (13). In addition, satellite imagery is available for the entire Amazon; see Fig. 1 for Google Earth Engine (GEE) examples used in the deforestation study of Bullock et al. (13). Angelopoulos et al. apply prediction-powered inference after using a portion of the labeled data and a gradient-boosted tree to fine-tune a regression-tree-based predictor of forest cover (14). Our work offers an alternative: We can avoid data splitting and instead apply cross-prediction, still with a gradient-boosted tree, to perform the fine-tuning. By doing so, we significantly reduce the size of the CI, as seen in Fig. 2. This trend will be consistent throughout our experiments: Cross-prediction is more efficient than prediction-powered inference with data splitting. Fig. 2 also shows that cross-prediction outperforms “classical” inference, which forms a CI based on gold-standard labels only and simply ignores the unlabeled data. Additional details about these experiments can be found in the Experiments section.

Another important takeaway from Fig. 2 is that cross-prediction gives more stable inferences: The confidence intervals have lower variability than the intervals computed via baseline approaches. Intuitively, classical inference has higher variability due to the smaller sample size, while prediction-powered inference has higher variability due to the arbitrariness in the data split. We will quantify the stability of cross-prediction in the Experiments section, showcasing its superiority across a range of examples; see Table 4.

Our work is also related to the literature known as semisupervised inference (15). The main difference between existing approaches and our work is that our proposal leverages black-box machine learning methods, allowing for more complicated data modalities (such as high-dimensional imagery) and more sophisticated ways of leveraging the unlabeled data. We elaborate on the relationship to prior work after introducing the formal problem setup.

## Problem Setup

We study statistical inference in a semisupervised setting, where collecting high-quality labels is challenging but feature observations are abundant. Formally, we have a dataset consisting of n i.i.d. feature–label pairs, $\left\{\left(X_{1},Y_{1}\right),\cdots ,\left(X_{n},Y_{n}\right)\right\}∼P^{n}$. In addition, we have a dataset consisting of N unlabeled data points, $\left\{{\tilde{X}}_{1},\cdots ,{\tilde{X}}_{N}\right\}∼P_{X}^{N}$, where $P_{X}$ denotes the marginal distribution over features according to P. We are most interested in the regime where N≫n, as in the case where feature collection is far cheaper than label collection.

Our goal is to perform inference on a property $\theta^{*}\left(P\right)$ of the data-generating distribution P, such as the mean outcome, a quantile of the outcome distribution, or a regression coefficient. Our proposal handles all estimands defined as a solution to an M-estimation problem:

[1] $$\begin{array}{c} \theta^{*}(\mathbb{P})=\underset{\theta }{\arg\min}\;L(\theta ),\;whereL(\theta ):=E\left[l_{\theta }(X,Y)\right], \end{array}$$

for a convex loss function $\ell_{\theta }$. Here and throughout, (X,Y) denotes a generic sample from P independent of everything else. All of the aforementioned estimands can be cast in the form (Eq. **1**). For example, if the target of inference is the mean outcome, $\theta^{*}\left(P\right)=E\left[Y\right]$, then $\theta^{*}\left(P\right)$ minimizes the squared loss:

[2] $$\begin{array}{c} \theta^{*}(\mathbb{P})=\underset{\theta }{\arg\min}\;E[l_{\theta }(Y)]=\underset{\theta }{\arg\min}E[{\left(Y-\theta \right)}^{2}]. \end{array}$$

Note that the estimand (and thus the loss) will sometimes only depend on a subset of the features X or only on the outcome Y, as in Eq. **2**. Also note that this manuscript focuses on $\theta^{*}\left(P\right)\in R^{d}$ for a fixed d. Studying high-dimensional settings—for example, understanding what scaling of d is permitted by the theory—is a valuable direction for future work. Below, we write $\theta^{*}=\theta^{*}\left(P\right)$ for short.

The main question we address is this: How should we leverage the unlabeled data to achieve both valid and powerful inference? Validity alone is an easy target: We can simply dispense with the unlabeled data and find the classical estimator ${\hat{\theta }}^{\mathrm{class}}$, defined as

[3] $${\hat{\theta }}^{\text{class}}\begin{array}{c} =\underset{\theta }{\arg\min}\;L^{\text{class}}(\theta ),\;\text{where}\;L^{\text{class}}(\theta ):=\frac{1}{n}\overset{n}{\underset{i=1}{\sum }}l_{\theta }(X_{i},Y_{i}). \end{array}$$

For all standard estimands defined via M-estimation—including means, quantiles, linear regression coefficients—there are off-the-shelf confidence intervals around ${\hat{\theta }}^{\mathrm{class}}$ that cover $\theta^{*}$ with a desired probability in the large-sample limit, see, e.g., refs. 16 and 17. The classical estimator and the corresponding CIs shall be the main comparison points used to evaluate the performance of cross-prediction.

## Related Work

We discuss the relationship between our work and the most closely related technical scholarship.

### Semisupervised Inference.

Our work falls within the literature known as semisupervised inference (15). Most existing work develops methods specialized to particular estimation problems, such as mean estimation (15, 18), quantile estimation (19), or linear regression (20, 21). One exception is the recent work of Song et al. (22), who also study general M-estimation. Their approach uses a projection-based correction to the classical loss (Eq. **3**) based on simple statistics from the unlabeled data, such as averages of low-degree polynomials of the features. Unlike existing proposals, our approach is based on imputing the missing labels using black-box machine learning methods, allowing for more complicated data modalities and more intricate ways of leveraging the unlabeled data. For example, it is unclear how to apply existing methods when the features $X_{i}$ are high-dimensional images. We also note that the semisupervised observation model has been long studied in semisupervised learning (23, 24). However, in this literature, the goal is prediction, rather than inference.

### Prediction-Powered Inference.

The core idea in this paper is to correct imputed predictions, and this derives from the proposal of prediction-powered inference (12). However, a key assumption in prediction-powered inference is that, in addition to a labeled and an unlabeled dataset, the analyst is given a good pretrained machine learning model. We make no such assumption. To apply the theory of prediction-powered inference, our setting would require using a portion of the labeled data for model training and leaving the rest for inference. In contrast, cross-prediction leverages each labeled data point for both model training and inference, leading to a boost in statistical power. The distinction between having and not having a pretrained model makes a difference even when comparing prediction-powered inference and the classical approach. Angelopoulos et al. (12) do not take into account the data used for model training when comparing the two baselines, because the model is assumed to have been trained before the analysis takes place. This makes sense when considering off-the-shelf models such as AlphaFold. In our comparisons, we do take the training data into account.

Angelopoulos et al. (25) show a central limit theorem for the prediction-powered estimator, allowing for computational and statistical improvements of the original methods for prediction-powered inference. Our inferences will be based on a similar central limit theorem for cross-prediction.

Wang et al. (26) similarly study inferences based on machine learning predictions. They propose using the labeled data to train a predictor of true outcomes from predicted ones, and then applying the predictor to debias the predictions on the unlabeled data. This algorithm does not come with a formal validity guarantee. Motwani and Witten (27) conduct a detailed empirical comparison of the method of Wang et al. and prediction-powered inference.

### Theory of Cross-Validation.

Cross-prediction is based on a form of cross-fitting. Consequently, our analysis is related to the theoretical studies of cross-validation (28–32). In particular, our theory borrows from the analysis of Bayle et al. (30), who prove a central limit theorem and study inference on the cross-validation test error. Our goal, however, is entirely different; we aim to provide inferential guarantees for an estimand $\theta^{*}$, as defined in Eq. **1**, in a semisupervised setting.

### Semiparametric Inference.

Our work is also related to the rich literature on semiparametric inference (33–40), where the goal is to do estimation in the presence of a high-dimensional nuisance parameter. Our debiasing strategy closely resembles doubly robust estimators (41), such as the AIPW estimator (42, 43), and one-step estimators (44). In this literature, the estimand is typically an expected value, such as the average treatment effect. One exception is the work of Jin and Rothenhäusler (45), who study general M-estimators through a semiparametric lens. The use of cross-fitting is common in that literature as well (40, 46, 47). While the technical arguments used in our work bear resemblance to those classically used in semiparametric inference, our motivation is different. Our focus is on showcasing how a theoretically principled use of black-box predictors—neural networks, random forests, and so on—on massive amounts of unlabeled data can boost inference. Since the practice of leveraging unlabeled data through predictions is already prevalent in domains such as remote sensing, our goal is to ground it in statistical theory.

### Inference with Missing Data.

Semisupervised inference can be seen as a special case of the problem of inference with missing data (48), where missing information about the labels occurs. Our proposed method bears similarities to multiple imputation (49–51) as, at least at a high level, it is based on “averaging out” multiple imputed predictions for the labels. However, our method is substantially different from multiple imputation, most notably due to the fact that it incorporates a particular form of debiasing to mitigate prediction inaccuracies.

### Inference under Model Misspecification.

Finally, our work relates to a large body of work on inference under model misspecification e.g., refs. 52–55. In particular, we do not assume that our predictions follow any “true” statistical model, and for parameters $\theta^{*}$ defined as a regression solution, we do not assume that the regression model is correct. For example, if $\theta^{*}$ is the solution to a linear regression, we do not assume that the data truly follows a linear model. Like in classical M-estimation, we will show asymptotic normality of our estimator despite the misspecification.

## Cross-Prediction

We propose cross-prediction—an estimation technique based on a combination of cross-fitting and prediction. The basic idea is to impute labels for the unlabeled data points, and then remove the bias arising from the inaccuracies in the predictions using the labeled data. We give a step-by-step outline of the construction of the cross-prediction estimator. In the following sections, we will show how to perform inference with this estimator; that is, how to perform hypothesis tests or construct confidence intervals for $\theta^{*}$.

### Cross-Prediction for Mean Estimation.

Before discussing the general case, we consider the problem of mean estimation to gain intuition; the object of inference is simply $\theta^{*}=E\left[Y\right]$.

We begin by partitioning the labeled dataset into K chunks, $I_{1}=\left\{1,\cdots ,n/K\right\},I_{2}=\left\{n/K+1,\cdots ,2n/K\right\}$, and so on (we assume for simplicity that n is divisible by K).^*^ Here, K is a user-specified number of folds, e.g., K=10. Then, as in cross-validation, we train a machine learning model K times, each time training on all data except one fold. Let $A_{\mathrm{train}}$ denote a possibly randomized training algorithm, which takes as input a dataset of arbitrary size and outputs a predictor of labels from features. Then, for each j∈[K], let $f^{\left(j\right)}$ be the model obtained by training on all folds but $I_{j}$; that is, $f^{\left(j\right)}=A_{\mathrm{train}}\left({\left\{\left(X_{i},Y_{i}\right)\right\}}_{i\in \left[n\right]\setminus I_{j}}\right)$. We note that $A_{\mathrm{train}}$ can be quite general; it may or may not treat the training data points symmetrically, and $f^{\left(j\right)}$ need not come from a well-defined family of predictors. Rather, $f^{\left(j\right)}$ can be any black-box model; e.g., a random forest, a gradient-boosted tree, a neural network, and so on. Moreover, $f^{\left(j\right)}$ can be trained from scratch or obtained by fine-tuning an off-the-shelf model. Finally, we use the trained models to impute predictions and compute the cross-prediction estimator, defined as

[4] $$\begin{array}{c} {\hat{\theta }}^{+}=\frac{1}{\mathrm{KN}}\sum_{j=1}^{K}\sum_{i=1}^{N}f^{\left(j\right)}\left({\tilde{X}}_{i}\right)-\frac{1}{n}\sum_{j=1}^{K}\sum_{i\in I_{j}}\left(f^{\left(j\right)}\left(X_{i}\right)-Y_{i}\right). \end{array}$$

Intuitively, the first term in (Eq. **4**) is an empirical approximation of the population mean if we treated the predictions as true labels. The second term in (Eq. **4**) serves to debias this heuristic: It subtracts an estimate of the bias between the predicted labels and the true labels. We note that the estimator (Eq. **4**) coincides with the mean estimator of Zhang and Bradic (18) in the special case where $f^{\left(j\right)}$ are linear models, that is, $f^{\left(j\right)}\left(x\right)=x^{⊤}\beta_{j}$ for some $\beta_{j}$. Our analysis applies more broadly, allowing for complex high-dimensional models (e.g., image classifiers).

Observe that the cross-prediction estimator is unbiased, i.e., $E\left[{\hat{\theta }}^{+}\right]=\theta^{*}$. Indeed, since $i\in I_{j}$ is not used to train model $f^{\left(j\right)}$, we have $E\left[f^{\left(j\right)}\left({\tilde{X}}_{i^{\prime }}\right)\right]=E\left[f^{\left(j\right)}\left(X_{i}\right)\right]$ for all $j\in \left[K\right],i\in I_{j},i^{\prime }\in \left[N\right]$. Applying this identity yields $E\left[{\hat{\theta }}^{+}\right]=E\left[Y\right]=\theta^{*}$.

The classical estimator is of course the sample mean:

[5] $$\begin{array}{c} {\hat{\theta }}^{\mathrm{class}}=\frac{1}{n}\sum_{i=1}^{n}Y_{i}, \end{array}$$

which is also unbiased. Given that both the cross-prediction estimator and the classical estimator are unbiased, it makes sense to ask which one has a lower variance. The main benefit of cross-prediction is that, if the trained models $f^{\left(j\right)}$ are reasonably accurate, we expect the variance of the cross-prediction estimator to be lower. To see this, first recall that, typically, N≫n. This means that the first term in ${\hat{\theta }}^{+}$ should have a vanishing variance due to the magnitude of N. Therefore,

$$\mathrm{Var}\left({\hat{\theta }}^{+}\right)\approx \mathrm{Var}\left(\frac{1}{n}\sum_{j=1}^{K}\sum_{i\in I_{j}}\left(f^{\left(j\right)}\left(X_{i}\right)-Y_{i}\right)\right).$$

As the sample mean, the remaining term is an average of n terms. However, when the models are accurate, i.e., $f^{\left(j\right)}\left(X_{i}\right)\approx Y_{i}$, we expect $\mathrm{Var}\left(f^{\left(j\right)}\left(X_{i}\right)-Y_{i}\right)\ll \mathrm{Var}\left(Y_{i}\right)$.

The closest alternative to the cross-prediction estimator is the prediction-powered estimator (12), that is, its straightforward adaptation to the setup without a pretrained model. As discussed earlier, prediction-powered inference relies on having a pretrained model f. We can reduce our setting to this case by introducing data splitting: We use the first $n_{\mathrm{tr}}\leq n$ data points from the labeled dataset to train a model f and the rest of the labeled data to compute the prediction-powered estimator:

[6] $$\begin{array}{c} {\hat{\theta }}^{\mathrm{PP}}=\frac{1}{N}\sum_{i=1}^{N}f\left({\tilde{X}}_{i}\right)-\frac{1}{n-n_{\mathrm{tr}}}\sum_{i=n_{\mathrm{tr}}+1}^{n}\left(f\left(X_{i}\right)-Y_{i}\right). \end{array}$$

The prediction-powered estimator is also unbiased: $E\left[{\hat{\theta }}^{\mathrm{PP}}\right]=\theta^{*}$. However, this strategy is potentially wasteful because, after f is trained, the training data are thrown away and not subsequently used for estimation. Cross-prediction uses the data more efficiently, by leveraging each data point for both training and estimation.

### General Cross-Prediction.

To introduce the cross-prediction estimator in full generality, recall that we are considering all estimands of the form (Eq. **1**). As in the case of mean estimation, we split the labeled data into K folds and train a predictive model $f^{\left(j\right)}$ on all folds but fold j∈[K]. The proposed cross-prediction estimator is defined as

[7] $$\begin{array}{c} \begin{array}{cc} & {\hat{\theta }}^{+}=\underset{\theta }{\arg\min}\;L^{+}\left(\theta \right),\;\text{where}\\ & L^{+}\left(\theta \right):=\frac{1}{\mathrm{KN}}\sum_{j=1}^{K}\sum_{i=1}^{N}{\tilde{\ell }}_{\theta ,i}^{f^{\left(j\right)}}-\frac{1}{n}\sum_{j=1}^{K}\sum_{i\in I_{j}}\left(\ell_{\theta ,i}^{f^{\left(j\right)}}-\ell_{\theta ,i}\right). \end{array} \end{array}$$

Here, we use the short-hand notation ${\tilde{\ell }}_{\theta ,i}^{f^{\left(j\right)}}:=\ell_{\theta }\left({\tilde{X}}_{i},f^{\left(j\right)}\left({\tilde{X}}_{i}\right)\right)$, $\ell_{\theta ,i}^{f^{\left(j\right)}}:=\ell_{\theta }\left(X_{i},f^{\left(j\right)}\left(X_{i}\right)\right)$, and $\ell_{\theta ,i}:=\ell_{\theta }\left(X_{i},Y_{i}\right)$. The intuition remains the same as before: The first term is an empirical approximation of the population loss if we treated the predictions as true labels, and the second term aims to debias this heuristic. One can verify that the mean estimator in (Eq. **4**) is indeed a special case of the general estimator in (Eq. **7**), by taking $\ell_{\theta }$ to be the squared loss, as per Eq. **2**.

The cross-prediction estimator optimizes an unbiased objective, since $E[L^{+}\left(\theta \right)]=L\left(\theta \right)$. This follows because $E\left[\ell_{\theta }\left({\tilde{X}}_{i^{\prime }},f^{\left(j\right)}\left({\tilde{X}}_{i^{\prime }}\right)\right)\right]=E\left[\ell_{\theta }\left(X_{i},f^{\left(j\right)}\left(X_{i}\right)\right)\right]$ for all $j\in \left[K\right],i\in I_{j},i^{\prime }\in \left[N\right]$, seeing that $i\in I_{j}$ is not used to train model $f^{\left(j\right)}$. Furthermore, by the same argument as before, we expect $L^{+}\left(\theta \right)$ to have a lower variance than the classical objective in (Eq. **3**) if N is large and the trained predictors are reasonably accurate. We note that $L^{+}\left(\theta \right)$ may not be a convex function in general, but solving for ${\hat{\theta }}^{+}$ is tractable in many cases of interest. For example, in the case of means and generalized linear models, $L^{+}\left(\theta \right)$ is convex.

The prediction-powered estimator is similar to the cross-prediction estimator, but it requires data splitting and does not average over multiple model fits. It is equal to

$$\begin{array}{c} \begin{array}{cc} & {\hat{\theta }}^{\mathrm{PP}}=\underset{\theta }{\arg\min}\;L^{\mathrm{PP}}\left(\theta \right),\;\text{where}\\ & L^{\mathrm{PP}}\left(\theta \right):=\frac{1}{N}\sum_{i=1}^{N}{\tilde{\ell }}_{\theta ,i}^{f}-\frac{1}{n-n_{\mathrm{tr}}}\sum_{i=n_{\mathrm{tr}}+1}^{n}\left(\ell_{\theta ,i}^{f}-\ell_{\theta ,i}\right), \end{array} \end{array}$$

where, as before, f is trained on the first $n_{\mathrm{tr}}$ labeled data points. The fact that cross-prediction averages the results of multiple model fits allows it to achieve more stable inference. Indeed, in our experiments, we will observe that cross-prediction is more stable than prediction-powered inference throughout.

## Inference for the Mean

We now discuss inference with the cross-prediction estimator. For simplicity, we first look at mean estimation, where $\theta^{*}=E\left[Y\right]$. We will see that much of the discussion will carry over to general M-estimation problems.

Inference with the cross-prediction estimator in (Eq. **4**) is difficult because the terms being averaged are all dependent through the labeled data. In contrast, the classical estimator in (Eq. **5**) averages independent terms, allowing for confidence intervals based on the central limit theorem. The prediction-powered estimator in (Eq. **6**) is similarly amenable to inference based on the central limit theorem, seeing that all the terms are independent conditional on f. In this section, we show that, under a relatively mild regularity condition, the cross-prediction estimator likewise satisfies a central limit theorem. This will in turn immediately allow constructing CIs and hypothesis tests for $\theta^{*}$.

The central limit theorem will require that, as the sample size grows, the predictions concentrate sufficiently rapidly around their expectation. Intuitively, one can think of the condition as requiring that the predictions are sufficiently stable. While the stability property is difficult to verify for complex black-box models, we empirically observe that inference based on the resulting central limit theorem nevertheless provides the correct coverage. We observe this across different estimation problems, data modalities, sample sizes, and so on.

Our analysis based on stability is inspired by the work of Bayle et al. (30), who study inference on the cross-validation test error, since the inferential challenges in cross-prediction are similar to those in cross-validation. The ultimate goals of the two analyses are, however, entirely different.

Below we state the stability condition. For all x, we define $\overset{¯}{f}\left(x\right):=E[f^{\left(1\right)}\left(x\right)]$; the “average” model $\overset{¯}{f}$ is the predictor we would obtain if we could train many models on independent datasets of size n−n/K and average out their predictions.

Assumption 1 We say that the predictions are stable if, as n→∞,

$$\sqrt{K\;\mathrm{Var}\left(f^{\left(1\right)}\left(X\right)-\overset{¯}{f}\left(X\right)|f^{\left(1\right)}\right)}\overset{L^{1}}{\to }0.$$

Assumption 1 requires that the models $f^{\left(j\right)}$ converge to their “average” model $\overset{¯}{f}$, but there is no assumption that $\overset{¯}{f}$ is by any means well-specified. If the number of folds is fixed (e.g., K=10), as we will typically assume, then Assumption 1 is satisfied if the variance of the difference between the learned predictions $f^{\left(1\right)}\left(X\right)$ and the average predictions $\overset{¯}{f}(X)$ vanishes at any rate, $\mathrm{Var}\left(f^{\left(1\right)}\left(X\right)-\overset{¯}{f}\left(X\right)|f^{\left(1\right)}\right)\overset{L^{1}}{\to }0$. We expect that any reasonably stable learning algorithm $A_{\mathrm{train}}$ should satisfy Assumption 1 (intuitively, any algorithm not too sensitive to perturbing a single data point). Violations of the assumption might arise if the number of folds is allowed to grow, e.g., as in the case of leave-one-out cross-fitting, since then the variance has to tend to zero sufficiently rapidly.

Equipped with Assumption 1, we can now state the central limit theorem for cross-prediction.

Theorem 1 **(Cross-prediction CLT for the mean).** Let $\theta^{*}$ be the mean outcome, $\theta^{*}=E\left[Y\right]$. Suppose that the predictions are stable (Assumption 1). Further, assume that $\frac{n}{N}$ has a limit, and that ${\overset{¯}{\sigma }}^{2}=\mathrm{Var}\left(\overset{¯}{f}\left(X\right)\right)$ and ${\overset{¯}{\sigma }}_{\Delta }^{2}=\mathrm{Var}\left(\overset{¯}{f}\left(X\right)-Y\right)$ have a nonzero limit. Then,

$$\frac{\sqrt{n}}{\sqrt{\frac{n}{N}{\overset{¯}{\sigma }}^{2}+{\overset{¯}{\sigma }}_{\Delta }^{2}}}\left({\hat{\theta }}^{+}-\theta^{*}\right)\overset{d}{\to }N\left(0,1\right).$$

With this, inference on $\theta^{*}$ is now straightforward as long as we can estimate the asymptotic variance consistently. We will discuss strategies for doing so later on.

Corollary 1 **(Cross-prediction inference for the mean).** Let $\theta^{*}$ be the mean outcome, $\theta^{*}=E\left[Y\right]$. Assume the conditions of Theorem 1, and suppose that we have estimators ${\hat{\sigma }}^{2}\overset{p}{\to }{\overset{¯}{\sigma }}^{2}$ and ${\hat{\sigma }}_{\Delta }^{2}\overset{p}{\to }{\overset{¯}{\sigma }}_{\Delta }^{2}$. Let

$$C_{\alpha }^{+}=\left({\hat{\theta }}^{+}\pm z_{1-\alpha /2}\frac{\sqrt{\frac{n}{N}{\hat{\sigma }}^{2}+{\hat{\sigma }}_{\Delta }^{2}}}{\sqrt{n}}\right).$$

Then, $\underset{n,N}{lim inf}\;P\left(\theta^{*}\in C_{\alpha }^{+}\right)\geq 1-\alpha .$

Per standard notation, $z_{1-\alpha /2}$ denotes the (1−α/2)-quantile of the standard normal distribution. Corollary 1 follows by a direct application of Theorem 1, together with Slutsky’s theorem.

## Inference for General M-Estimation

We generalize the principle introduced in the last section to handle arbitrary M-estimation problems. Indeed, the results presented in this section will strictly subsume the previous results.

As in the case of the mean, we will require that the predictions are “stable” in an appropriate sense. Naturally, the notion of stability will depend on the loss function used to define the M-estimator.

Assumption 2 With $\overset{¯}{f}(\cdot )$ as before, we say that the predictions are stable if for all θ, as n→∞,

$$\sqrt{K\;\mathrm{Var}\left(\nabla \ell_{\theta }\left(X,f^{\left(1\right)}\left(X\right)\right)-\nabla \ell_{\theta }\left(X,\overset{¯}{f}\left(X\right)\right)|f^{\left(1\right)}\right)}\overset{L^{1}}{\to }0.$$

Here, $\mathrm{Var}(\cdot |f^{\left(1\right)})$ denotes the covariance matrix conditional on $f^{\left(1\right)}$. Also, for vectors and matrices, by “$\overset{L^{1}}{\to }0$” we mean convergence in mean to zero element-wise. Notice that by setting $\ell_{\theta }\left(y\right)={\left(\theta -y\right)}^{2}$ to be the squared loss, Assumption 2 reduces to Assumption 1 in the case of mean estimation. As in the case of Assumption 1, Assumption 2 should be interpreted as a stability requirement on $A_{\mathrm{train}}$. Moreover, there is again no requirement of correct specification of $\overset{¯}{f}$.

We will provide two approaches to inference in this section; which one is more appropriate will depend on the inference problem at hand.

One approach will be based on the characterization of $\theta^{*}$ as a zero of the gradient of the expected loss, $\nabla L\left(\theta^{*}\right)=E\left[\nabla \ell_{\theta^{*}}\left(X,Y\right)\right]=0$, which follows by the convexity of the loss. In particular, we will construct a confidence set for $\theta^{*}$ by finding all θ accepted by a valid test for the null hypothesis that ∇L(θ)=0. Since the test is valid and $\theta^{*}$ satisfies the null condition, the true solution $\theta^{*}$ will be excluded with small probability. The hypothesis test for the population gradient ∇L(θ) will follow from a central limit theorem for the gradient of the cross-prediction loss,

$$\nabla L^{+}\left(\theta \right)=\frac{1}{\mathrm{KN}}\sum_{j=1}^{K}\sum_{i=1}^{N}\nabla {\tilde{\ell }}_{\theta ,i}^{f^{\left(j\right)}}-\frac{1}{n}\sum_{j=1}^{K}\sum_{i\in I_{j}}\left(\nabla \ell_{\theta ,i}^{f^{\left(j\right)}}-\nabla \ell_{\theta ,i}\right).$$

The other approach will be based on showing asymptotic normality of the cross-prediction estimator. For this, we build on the proof of asymptotic normality of the prediction-powered estimator (with a pretrained model) (25), which in turn builds on classical asymptotic normality of M-estimators (17). The asymptotic normality will allow forming standard CLT intervals around ${\hat{\theta }}^{+}$.

We implicitly assume mild regularity on the losses $\ell_{\theta }\left(x,y\right)$ and $\ell_{\theta }\left(x,f^{\left(j\right)}\left(x\right)\right)$, in particular that they are differentiable and locally Lipschitz around $\theta^{*}$ for all possible $f^{\left(j\right)}$ (see definition A.1 in ref. 25). Our second inference approach will require the usual condition that ${\hat{\theta }}^{+}$ is consistent, ${\hat{\theta }}^{+}\overset{p}{\to }\theta^{*}$; this is satisfied quite broadly, e.g., when the parameter space is compact or when $L^{+}\left(\theta \right)$ is convex. The latter holds for all generalized linear models, for example. See refs. 17 and 25 for further discussion.

Theorem 2 states the main technical result of this section, which extends Theorem 1 to general M-estimation problems.

Theorem 2 **(Cross-prediction CLT).** Suppose that the predictions are stable (Assumption 2). Further, assume that $\frac{n}{N}$ has a limit, and that ${\overset{¯}{\Sigma }}_{\theta }=\mathrm{Var}\left(\nabla \ell_{\theta ,i}^{\overset{¯}{f}}\right)$ and ${\overset{¯}{\Sigma }}_{\Delta ,\theta }=\mathrm{Var}\left(\nabla \ell_{\theta ,i}^{\overset{¯}{f}}-\nabla \ell_{\theta ,i}\right)$ have a nonzero limit. Denote ${\overset{¯}{V}}_{\theta }=\frac{n}{N}{\overset{¯}{\Sigma }}_{\theta }+{\overset{¯}{\Sigma }}_{\Delta ,\theta }$. Then,

$$\sqrt{n}{\overset{¯}{V}}_{\theta }^{-1/2}\left(\nabla L^{+}\left(\theta \right)-\nabla L\left(\theta \right)\right)\overset{d}{\to }N\left(0,I\right).$$

If, additionally, the Hessian $H_{\theta^{*}}=\nabla^{2}L\left(\theta^{*}\right)$ is nonsingular, ${\hat{\theta }}^{+}\overset{p}{\to }\theta^{*}$, and K is constant, then

$$\sqrt{n}{\overset{¯}{\Sigma }}^{-1/2}\left({\hat{\theta }}^{+}-\theta^{*}\right)\overset{d}{\to }N\left(0,I\right),$$

where $\overset{¯}{\Sigma }=H_{\theta^{*}}^{-1}{\overset{¯}{V}}_{\theta^{*}}H_{\theta^{*}}^{-1}$.

Theorem 2 immediately yields two methods for computing a confidence set for $\theta^{*}$, as stated below.

Corollary 2 **(Cross-prediction inference).** Suppose that we have estimators $\hat{\Sigma }\overset{p}{\to }\overset{¯}{\Sigma }$ and ${\hat{V}}_{\theta }\overset{p}{\to }{\overset{¯}{V}}_{\theta }$, for all θ. Then, assuming the conditions of Theorem 2, for either

$$\begin{array}{cc} & C_{\alpha }^{+}=\left\{\theta :{∥{\hat{V}}_{\theta }^{-1/2}\nabla L^{+}\left(\theta \right)∥}^{2}\leq \frac{\chi_{d,1-\alpha }^{2}}{n}\right\}\;\text{or}\\ & C_{\alpha }^{+}={\left({\hat{\theta }}_{i}^{+}\pm z_{1-\alpha /(2d)}\sqrt{\frac{{\hat{\Sigma }}_{\mathrm{ii}}}{n}},\right)}_{i=1}^{d}, \end{array}$$

we have $\underset{n,N}{lim inf}\;P\left(\theta^{*}\in C_{\alpha }^{+}\right)\geq 1-\alpha .$

Above, $\chi_{d,1-\alpha }^{2}$ is the (1−α)-quantile of the chi-squared distribution with d degrees of freedom; when d=1 (as in the case of mean estimation), $\chi_{d,1-\alpha }$ is equal to $z_{1-\alpha /2}$. Note also that in the case of mean estimation, the two confidence sets are identical and reduce to the set from Corollary 1. In the second confidence set we apply a Bonferroni correction over the d coordinates of the estimand for simplicity and clarity of exposition; we can obtain an asymptotically exact (1−α)-confidence set as $C_{\alpha }^{+}=\left\{{\hat{\theta }}^{+}+v:v^{⊤}\hat{\Sigma }v\leq \frac{\chi_{d,1-\alpha }^{2}}{n}\right\}$.

Next, we apply Theorem 2 and Corollary 2 to concrete problems—quantile estimation, linear regression, and generalized linear models—to get explicit CI constructions.

### Example: Quantile Estimation.

Assume we are interested in a quantile of Y, $\theta^{*}=\inf\left\{y:P(Y\leq y)\geq q\right\}.$ The quantile can equivalently be written as any minimizer of the pinball loss,

$$\begin{array}{cc} \theta^{*} & =\underset{\theta }{\arg\min}\;E\left[\ell_{\theta }\left(Y\right)\right]\\ & =\underset{\theta }{\arg\min}\;E\left[q\left(Y-\theta \right)1\left\{Y>\theta \right\}+\left(1-q\right)\left(\theta -Y\right)1\left\{Y\leq \theta \right\}\right]. \end{array}$$

The subgradient of the pinball loss is equal to $\nabla \ell_{\theta }\left(y\right)=-q1\left\{y>\theta \right\}+\left(1-q\right)1\left\{y\leq \theta \right\}=-q+1\left\{y\leq \theta \right\}$. Plugging this expression into the first confidence set from Corollary 2 yields

$$C_{\alpha }^{+}=\left\{\theta :\left|{\tilde{F}}^{+}\left(\theta \right)-\Delta^{+}\left(\theta \right)-q\right|\leq z_{1-\alpha /2}\frac{\sqrt{\frac{n}{N}{\hat{\sigma }}_{\theta }^{2}+{\hat{\sigma }}_{\Delta ,\theta }^{2}}}{\sqrt{n}}\right\},$$

where ${\tilde{F}}^{+}\left(\theta \right)=\frac{1}{\mathrm{KN}}\sum_{j=1}^{K}\sum_{i=1}^{N}1\left\{f^{\left(j\right)}\left({\tilde{X}}_{i}\right)\leq \theta \right\}$ is the average empirical CDF of the predictions on the unlabeled data, and $\Delta^{+}\left(\theta \right)=\frac{1}{n}\sum_{j=1}^{K}\sum_{i\in I_{j}}\left(1\left\{f^{\left(j\right)}\left(X_{i}\right)\leq \theta \right\}-1\left\{Y_{i}\leq \theta \right\}\right)$ is the difference between the empirical CDFs of the predictions and true outcomes on the labeled data. The SEs are equal to ${\overset{¯}{\sigma }}_{\theta }^{2}=\mathrm{Var}\left(1\left\{\overset{¯}{f}\left(X\right)\leq \theta \right\}\right)$ and ${\overset{¯}{\sigma }}_{\Delta ,\theta }^{2}=\mathrm{Var}\left(1\left\{\overset{¯}{f}\left(X\right)\leq \theta \right\}-1\left\{Y\leq \theta \right\}\right)$. The confidence set $C_{\alpha }^{+}$ thus consists of all values θ such that the average predicted CDF ${\tilde{F}}^{+}\left(\theta \right)$, corrected by the bias $\Delta^{+}\left(\theta \right)$, is close to the target level q.

### Example: Linear Regression.

In linear regression, the target of inference is defined by

[8] $$\begin{array}{c} \theta^{*}=\underset{\theta }{\arg\min}\;E\left[\ell_{\theta }\left(X,Y\right)\right]=\underset{\theta }{\arg\min}\;\frac{1}{2}E\left[{\left(Y-X^{⊤}\theta \right)}^{2}\right]. \end{array}$$

In this case, the cross-prediction estimator, equal to the solution to $\nabla L^{+}\left({\hat{\theta }}^{+}\right)=0$, has a closed-form expression. Letting $\tilde{X}\in R^{N\times d}$ (resp. $X\in R^{n\times d}$) be the unlabeled (resp. labeled) data matrix, $Y\in R^{n}$ be the vector of labeled outcomes, the solution is given by

$$\begin{array}{c} {\hat{\theta }}^{+}={\left({\tilde{X}}^{⊤}\tilde{X}\right)}^{-1}\left({\tilde{X}}^{⊤}f_{\mathrm{avg}(K)}\left(\tilde{X}\right)-\frac{N}{n}\cdot X^{⊤}\left(f_{1:K}\left(X\right)-Y\right)\right), \end{array}$$

where $f_{\mathrm{avg}(K)}\left(\tilde{X}\right)=\frac{1}{K}\sum_{j=1}^{K}f^{\left(j\right)}\left(\tilde{X}\right)$ is the vector of average predictions on the unlabeled data, and $f_{1:K}\left(X\right)$ is the vector of predictions on the labeled data: $f_{1:K}\left(X\right)=\left(f^{\left(1\right)}\left(X_{1}\right),\cdots ,f^{\left(1\right)}\left(X_{\frac{n}{K}}\right),f^{\left(2\right)}\left(X_{\frac{n}{K}+1}\right),\cdots ,f^{\left(K\right)}\left(X_{n}\right)\right)$. We see that ${\hat{\theta }}^{+}$ resembles the usual least-squares estimator with $f_{\mathrm{avg}(K)}\left(\tilde{X}\right)$ as the response, except for the extra debiasing factor, $\frac{N}{n}\cdot X^{⊤}\left(f_{1:K}\left(X\right)-Y\right)$, that takes into account the prediction inaccuracies.

Instantiating the relevant terms, Theorem 2 implies that ${\hat{\theta }}^{+}$ is asymptotically normal with covariance equal to $\Sigma_{\mathrm{OLS}}=H^{-1}{\overset{¯}{V}}_{\theta^{*}}H^{-1}$, where $H=E[XX^{⊤}]$ and ${\overset{¯}{V}}_{\theta^{*}}=\frac{n}{N}{\overset{¯}{\Sigma }}_{\theta^{*}}+{\overset{¯}{\Sigma }}_{\Delta }$, for ${\overset{¯}{\Sigma }}_{\theta^{*}}=\mathrm{Var}\left(\left(\overset{¯}{f}\left(X\right)-X^{⊤}\theta^{*}\right)X\right)$ and ${\overset{¯}{\Sigma }}_{\Delta }=\mathrm{Var}\left(\left(\overset{¯}{f}\left(X\right)-Y\right)X\right)$.

For a given coordinate of interest i, a CI for $\theta_{i}^{*}$ can therefore be obtained as

$$C_{\alpha }^{+}=\left({\hat{\theta }}_{i}^{+}\pm z_{1-\alpha /2}\frac{\sqrt{{\left({\hat{\Sigma }}_{\mathrm{OLS}}\right)}_{\mathrm{ii}}}}{\sqrt{n}}\right),$$

given an estimate ${\hat{\Sigma }}_{\mathrm{OLS}}$ of $\Sigma_{\mathrm{OLS}}$.

### Example: Generalized Linear Models.

We can generalize the previous example by considering all generalized linear models (GLMs). In particular, we consider targets of inference given by

[9] $$\begin{array}{cc} \theta^{*} & =\underset{\theta }{\arg\min}\;E\left[-\log p_{\theta }\left(Y|X\right)\right]\\ & =\underset{\theta }{\arg\min}\;E\left[-Y\theta^{⊤}X+\psi \left(X^{⊤}\theta \right)\right], \end{array}$$

where $p_{\theta }\left(y|x\right)=\;\exp\left(yx^{⊤}\theta -\psi \left(x^{⊤}\theta \right)\right)$ is the probability density of the outcome given the features and the log-partition function ψ is convex. The objective (Eq. **9**) recovers the linear-regression objective (Eq. **8**) by setting $\psi \left(s\right)=\frac{1}{2}s^{2}$. It captures logistic regression by choosing $\psi \left(s\right)=\;\log\left(1+e^{s}\right)$.

The asymptotic covariance given by Theorem 2 evaluates to $\Sigma_{\mathrm{GLM}}=H_{\theta^{*}}^{-1}{\overset{¯}{V}}_{\theta^{*}}H_{\theta^{*}}^{-1}$, $H_{\theta^{*}}=E\left[\psi^{\prime \prime }\left(X^{⊤}\theta^{*}\right)XX^{⊤}\right]$, ${\overset{¯}{V}}_{\theta^{*}}=\frac{n}{N}\mathrm{Var}\left(\left(\psi^{\prime }\left(X^{⊤}\theta^{*}\right)-\overset{¯}{f}\left(X\right)\right)X\right)+\mathrm{Var}\left(\left(\overset{¯}{f}\left(X\right)-Y\right)X\right)$. Therefore, analogously to the OLS case, given an estimate ${\hat{\Sigma }}_{\mathrm{GLM}}$ of $\Sigma_{\mathrm{GLM}}$, we can construct a CI for ${\hat{\theta }}^{+}$ as

$$C_{\alpha }^{+}=\left({\hat{\theta }}_{i}^{+}\pm z_{1-\alpha /2}\frac{\sqrt{{\left({\hat{\Sigma }}_{\mathrm{GLM}}\right)}_{\mathrm{ii}}}}{\sqrt{n}}\right).$$

## Variance Estimation via Bootstrapping

The previous inference results rely on being able to estimate the asymptotic covariance of ${\hat{\theta }}^{+}$. We herewith provide an explicit estimation strategy that we will use in our experiments.

Recall that the asymptotic covariance is equal to $\overset{¯}{\Sigma }=H_{\theta^{*}}^{-1}{\overset{¯}{V}}_{\theta^{*}}H_{\theta^{*}}^{-1}$, where ${\overset{¯}{V}}_{\theta }=\frac{n}{N}{\overset{¯}{\Sigma }}_{\theta }+{\overset{¯}{\Sigma }}_{\Delta ,\theta }$, for ${\overset{¯}{\Sigma }}_{\theta }=\mathrm{Var}\left(\nabla \ell_{\theta ,i}^{\overset{¯}{f}}\right)$ and ${\overset{¯}{\Sigma }}_{\Delta ,\theta }=\mathrm{Var}\left(\nabla \ell_{\theta ,i}^{\overset{¯}{f}}-\nabla \ell_{\theta ,i}\right)$. Estimating the Hessian $H_{\theta }$ is easy via plug-in estimation; ${\overset{¯}{V}}_{\theta }$, on the other hand, depends on the average model $\overset{¯}{f}$. If the average model $\overset{¯}{f}$ was known, one could compute estimates of ${\overset{¯}{\Sigma }}_{\theta }$ and ${\overset{¯}{\Sigma }}_{\Delta ,\theta }$ by replacing the true covariances with their empirical counterparts. Thus, the challenge is to approximate $\overset{¯}{f}$. To achieve this, we apply the bootstrap to simulate multiple model training runs, and at the end, we average the predictions of all the learned models.

In more detail, for each b∈{1,2,…,B}=[B], we sample $n-\frac{n}{K}$ data points uniformly at random from the labeled dataset, and denote the indices of the samples by $I_{\mathrm{boot}}^{b}$. Then, we use the sampled data points to train a model $f_{\mathrm{boot}}^{\left(b\right)}$ using the same model-fitting strategy as for the cross-prediction models $f^{\left(j\right)}$. To estimate ${\overset{¯}{\Sigma }}_{\theta }$, we compute

$${\hat{\Sigma }}_{\theta }=\hat{\mathrm{Var}}\left(\nabla \ell_{\theta }\left({\tilde{X}}_{i},{\overset{¯}{f}}_{\mathrm{boot}}\left({\tilde{X}}_{i}\right)\right),i\in \left[N\right]\right),$$

where ${\overset{¯}{f}}_{\mathrm{boot}}=\frac{1}{B}\sum_{b=1}^{B}f_{\mathrm{boot}}^{\left(b\right)}$ and $\hat{\mathrm{Var}}$ denotes the empirical covariance. To estimate ${\overset{¯}{\Sigma }}_{\Delta ,\theta }$, we compute

$${\hat{\Sigma }}_{\Delta ,\theta }=\hat{\mathrm{Var}}\left({\left(\nabla \ell_{\theta }\left(X_{i},f_{\mathrm{boot}}^{\left(b\right)}\left(X_{i}\right)\right)-\nabla \ell_{\theta }\left(X_{i},Y_{i}\right)\right)}_{i\in \left[n\right]\setminus I_{\mathrm{boot}}^{\left(b\right)},b\in \left[B\right]}\right).$$

Finally, we approximate the covariance by $\frac{n}{N}{\hat{\Sigma }}_{\theta }+{\hat{\Sigma }}_{\Delta ,\theta }$. In computing ${\hat{\Sigma }}_{\Delta ,\theta }$, we technically do not average out the bootstrapped models because we want to make sure that every point $\left(X_{i},Y_{i}\right)$ used to compute the gradient bias is independent of its corresponding model $f_{\mathrm{boot}}^{\left(b\right)}$. Intuitively, as per the discussion following Assumption 1, if $A_{\mathrm{train}}$ is stable we expect $f_{\mathrm{boot}}^{\left(b\right)}$ to be a good approximation of the average model $\overset{¯}{f}$, which in turn means that the bootstrap covariance estimates should be consistent per conventional wisdom. We show empirically that the covariance estimates lead to valid coverage across a range of applications.

To give one concrete example, consider mean estimation: $\theta^{*}=E\left[Y\right]$. We compute

$$\begin{array}{cc} & {\hat{\sigma }}^{2}=\hat{\mathrm{Var}}\left({\overset{¯}{f}}_{\mathrm{boot}}\left({\tilde{X}}_{i}\right),i\in \left[N\right]\right)\;\text{and}\\ & {\hat{\sigma }}_{\Delta }^{2}=\hat{\mathrm{Var}}\left(f_{\mathrm{boot}}^{\left(b\right)}\left(X_{i}\right)-Y_{i},i\in \left[n\right]\setminus I_{\mathrm{boot}}^{\left(b\right)},b\in \left[B\right]\right), \end{array}$$

and take $C_{\alpha }^{+}=\left({\hat{\theta }}^{+}\pm z_{1-\alpha /2}\frac{\sqrt{\frac{n}{N}{\hat{\sigma }}^{2}+{\hat{\sigma }}_{\Delta }^{2}}}{\sqrt{n}}\right)$ as the final interval.

## Experiments

We evaluate cross-prediction and compare it to baseline approaches on several datasets; the baselines are the classical inference method, which only uses the labeled data, and prediction-powered inference with a data-splitting step in order to train a predictive model. Code for reproducing the experiments is available at: https://github.com/tijana-zrnic/cross-ppi (56).

For each experimental setting, we plot the coverage and CI width estimated over 100 trials for all baselines. We also show the constructed CIs for five randomly chosen trials. Finally, to quantify the stability of inferences, we report the SD of the lower and upper endpoints of the confidence intervals for each method.

We begin with proof-of-concept experiments on synthetic data. Then, we move on to more complex real datasets.

### Proof-of-Concept Experiments on Synthetic Data.

To build intuition, we begin with simple experiments on synthetic data. The purpose is to confirm what we expect in theory: a) as it gets easier to predict labels from features, cross-prediction, and prediction-powered inference become more powerful and increasingly outperform the classical approach; b) cross-prediction uses the data more efficiently than prediction-powered inference, yielding smaller intervals; c) cross-prediction gives more stable inferences than the baselines when the predictions are useful; d) all three approaches lead to satisfactory coverage.

In all of the following experiments, we have N=10,000 unlabeled data points, and we vary the size of the labeled data n between 100 and 1,000, in 100-point increments. We apply cross-prediction with K=10 folds. We estimate the variance using the bootstrap approach described in the last section, with B=30 bootstrap samples. For prediction-powered inference, we use half of the labeled data for model training. To illustrate the point that cross-prediction can be applied with any black-box model, we train gradient-boosted trees via XGBoost (57) to obtain the models $f^{\left(j\right)}$. We use the same model-fitting strategy for prediction-powered inference. We fix the target error level to be α=0.1 and average the results over 100 trials.

#### Mean estimation.

For given parameters $R^{2}$ and $\sigma_{Y}^{2}$, the data-generating distribution is defined as $X∼N\left(0,I_{2}\right),Y=\mu +X^{⊤}\beta +\xi$, where $\beta_{1}=\beta_{2}=R\sigma_{Y}/\sqrt{2}$, and $\xi ∼N(0,\sigma_{Y}^{2}\left(1-R^{2}\right))$ is independent of X. We fix $\mu =4,\sigma_{Y}^{2}=4$ and vary $R^{2}=\frac{\mathrm{Var}(X^{⊤}\beta )}{\mathrm{Var}(Y)}\in \left\{0,0.5,1\right\}$. The idea is to vary the degree to which the outcomes can be explained through the features: When $R^{2}=0$, the outcome is independent of the features and we do not expect predictions to help, while when $R^{2}=1$, the outcome can be perfectly explained through the features and we expect predictions to be helpful. Given that the variance of Y is kept constant regardless of $R^{2}$, classical inference has the same distribution of widths across $R^{2}$. The target of inference is $\theta^{*}=E\left[Y\right]=\mu$.

**Fig. 3.**
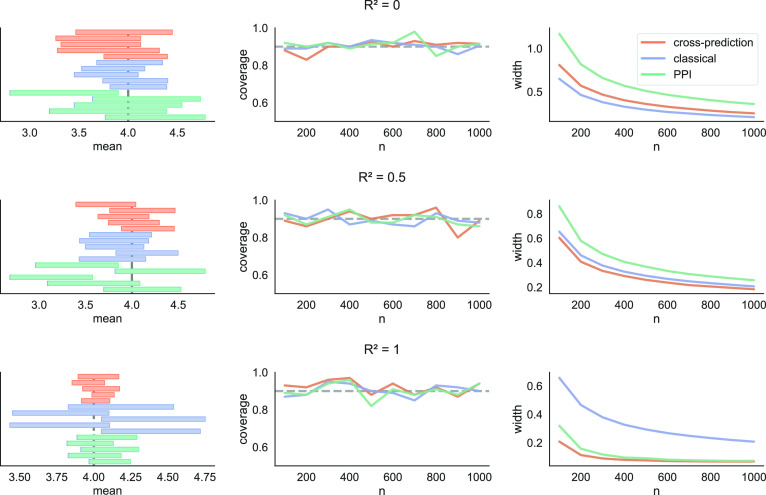
Mean estimation. Intervals from five randomly chosen trials (*Left*), coverage (*Middle*), and average interval width (*Right*) of cross-prediction, classical inference, and prediction-powered inference (PPI) in a mean estimation problem.

In Fig. 3 we plot the coverage and interval width of cross-prediction, classical inference, and prediction-powered inference, as well as five example intervals. All three methods approximately achieve the target coverage, and cross-prediction dominates prediction-powered inference throughout. Further, we see that the classical approach dominates the alternatives when the features are independent of the outcomes, while the alternatives become more powerful as $R^{2}$ increases. To evaluate stability, in Table 1 we report the SD of the lower and upper endpoints of the confidence intervals from Fig. 3, for n=100. We observe that the classical approach is the most stable method when $R^{2}=0$, which makes sense because the predictions can only introduce noise. When $R^{2}=0.5$, cross-prediction and classical inference have a similar degree of stability, while when $R^{2}=1$ cross-prediction is significantly more stable. Moreover, cross-prediction is significantly more stable than prediction-powered inference throughout. These trends hold across different values of n; however, we only include the results for n=100 for simplicity of exposition.

**Table 1.** SD of the lower (${\hat{\sigma }}_{L}$) and upper (${\hat{\sigma }}_{U}$) endpoints of the CIs in the mean estimation problem from Fig. 3, for n=100

	Mean estimation	
	$R^{2}=0$	
Method	${\hat{\sigma }}_{L}$	${\hat{\sigma }}_{U}$
Cross-prediction	0.2694	0.2696
Classical	0.2124	0.2085
PPI	0.3844	0.3997
	$R^{2}=0.5$	
Method	${\hat{\sigma }}_{L}$	${\hat{\sigma }}_{U}$
Cross-prediction	0.1769	0.1897
Classical	0.1908	0.1885
PPI	0.2751	0.2684
	$R^{2}=1$	
Method	${\hat{\sigma }}_{L}$	${\hat{\sigma }}_{U}$
Cross-prediction	0.0591	0.0613
Classical	0.2136	0.2102
PPI	0.1045	0.1061

The minimum value in each column is in bold.

#### Quantile estimation.

We adopt the same data-generating process as for mean estimation. We only change the target of inference $\theta^{*}$ to be the 75th percentile of the outcome distribution.

**Fig. 4.**
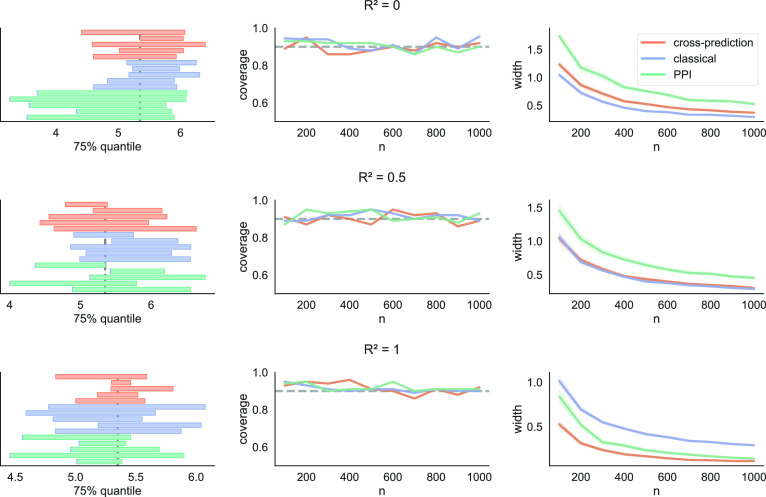
Quantile estimation. Intervals from five randomly chosen trials (*Left*), coverage (*Middle*), and average interval width (*Right*) of cross-prediction, classical inference, and prediction-powered inference (PPI) in a quantile estimation problem. The target is the 75th percentile.

In Fig. 4 we plot the coverage and interval width of cross-prediction, classical inference, and prediction-powered inference, as well as five example intervals. We observe a qualitatively similar comparison as in the case of mean estimation: All three methods approximately achieve the target coverage, and cross-prediction dominates prediction-powered inference throughout. As before, the classical approach dominates the alternatives when the features are independent of the outcomes, and the alternatives become increasingly powerful as $R^{2}$ increases. In Table 2 we evaluate the stability of the methods by reporting the SD of the lower and upper endpoints of the confidence intervals from Fig. 4, for n=100. As before, Table 2 shows that cross-prediction is more stable than prediction-powered inference for all values of $R^{2}$, and when $R^{2}=0$ classical inference is the most stable option. When $R^{2}=0.5$, cross-prediction has a slightly more stable upper endpoint than classical inference, while classical inference has a more stable lower endpoint. When $R^{2}=1$, cross-prediction is by far the most stable method. For $R^{2}\in \left\{0,0.5\right\}$. Again, these trends are largely consistent across different values of n; however, we only include the results for n=100 for simplicity.

**Table 2.** SD of the lower (${\hat{\sigma }}_{L}$) and upper (${\hat{\sigma }}_{U}$) endpoints of the CIs in the quantile estimation problem from Fig. 4, for n=100

	Quantile estimation	
	$R^{2}=0$	
Method	${\hat{\sigma }}_{L}$	${\hat{\sigma }}_{U}$
Cross-prediction	0.4102	0.3509
Classical	0.2302	0.3024
PPI	0.5424	0.4614
	$R^{2}=0.5$	
Method	${\hat{\sigma }}_{L}$	${\hat{\sigma }}_{U}$
Cross-prediction	0.3253	0.3242
Classical	0.2569	0.3305
PPI	0.4141	0.4368
	$R^{2}=1$	
Method	${\hat{\sigma }}_{L}$	${\hat{\sigma }}_{U}$
Cross-prediction	0.1345	0.1545
Classical	0.2615	0.2806
PPI	0.2151	0.3280

The minimum value in each column is in bold.

#### Linear regression.

Finally, we look at linear regression. For robustness and interpretability, it is common to include only a subset of the available features in the regression. The process of deciding which variables to include is known as model selection. The variables that are not included in the model may still be predictive of the outcome of interest; we demonstrate that, as such, they can be useful for inference.

The data-generating distribution is defined as follows: We generate $X∼N(0,I_{3})$, $Y=X^{⊤}\beta +\xi$, where $\beta =(1,1,R_{0}\sigma_{Y})$ and $\xi ∼N(0,\sigma_{Y}^{2}\left(1-R_{0}^{2}\right))$. Again, the idea is to vary how much of the outcome can be explained through prediction versus how much of it is exogenous randomness. We fix $\sigma_{Y}^{2}=4$ and vary $R_{0}\in \left\{0,0.5,1\right\}$. The target of inference is defined as the least-squares solution when regressing Y on $\left(X_{1},X_{2}\right)$, that is, the first coordinate of this solution. In this case, this is simply equal to $\theta^{*}=\beta_{1}=1$.

**Fig. 5.**
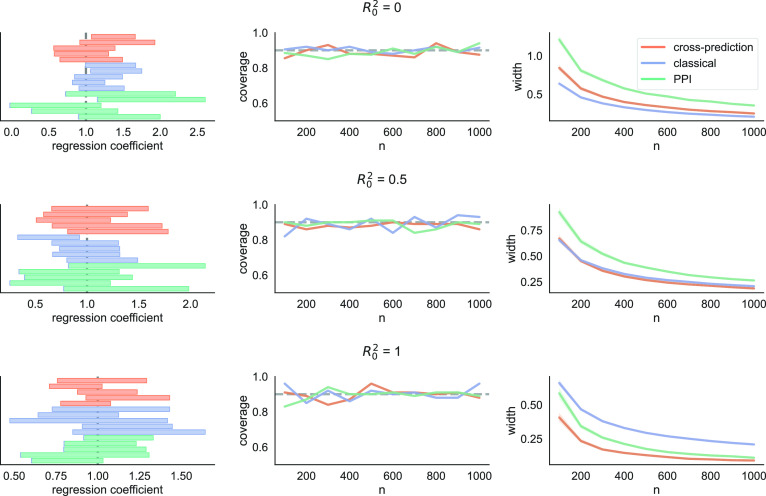
Linear regression. Intervals from five randomly chosen trials (*Left*), coverage (*Middle*), and average interval width (*Right*) of cross-prediction, classical inference, and prediction-powered inference (PPI) in a linear regression problem.

In Fig. 5 we plot the coverage and interval width of cross-prediction, classical inference, and prediction-powered inference. When $R_{0}^{2}=0$, the classical approach outperforms the prediction-based approaches; as $R_{0}^{2}$ grows, meaning more of the randomness of the outcome can be attributed to $X_{3}$, the prediction-based approaches dominate the classical one. As before, cross-prediction yields smaller intervals than prediction-powered inference. We remark that, even though the inference problem posits a linear model, the prediction-based approaches still use XGBoost for model training. Like in the previous two examples, we report on the stability of the three methods in Table 3. We again fix n=100 for simplicity. Cross-prediction is far more stable than prediction-powered inference throughout, and it is more stable than classical inference for nonzero values of $R_{0}^{2}$.

**Table 3.** SD of the lower (${\hat{\sigma }}_{L}$) and upper (${\hat{\sigma }}_{U}$) endpoints of the CIs in the linear regression problem from Fig. 5, for n=100

	Linear regression	
	$R_{0}^{2}=0$	
Method	${\hat{\sigma }}_{L}$	${\hat{\sigma }}_{U}$
Cross-prediction	0.2801	0.2969
Classical	0.2091	0.2098
PPI	0.4104	0.4870
	$R_{0}^{2}=0.5$	
Method	${\hat{\sigma }}_{L}$	${\hat{\sigma }}_{U}$
Cross-prediction	0.1875	0.2250
Classical	0.2271	0.2262
PPI	0.2602	0.3326
	$R_{0}^{2}=1$	
Method	${\hat{\sigma }}_{L}$	${\hat{\sigma }}_{U}$
Cross-prediction	0.1102	0.1472
Classical	0.1800	0.1809
PPI	0.1522	0.2530

The minimum value in each column is in bold.

### Estimating Deforestation from Satellite Imagery.

We briefly revisit the problem of deforestation analysis from Fig. 2. As we saw in the figure, cross-prediction gave tighter CIs for the deforestation rate than using gold-standard measurements of deforestation alone. In other words, cross-prediction can enable a reduction in the number of necessary field visits to measure deforestation. Moreover, we saw that cross-prediction outperformed prediction-powered inference.

Here we argue another benefit of cross-prediction in this problem: It is a more stable solution than the baselines. Table 4 shows the SD of the endpoints of the confidence intervals constructed by cross-prediction and its competitors. Cross-prediction has a significantly lower variability of the endpoints than both classical inference and prediction-powered inference, while the latter two exhibit similar variability.

**Table 4.** SD of the lower (${\hat{\sigma }}_{L}$) and upper (${\hat{\sigma }}_{U}$) endpoints of the CIs in the real-data problems

	Deforestation analysis	
Method	${\hat{\sigma }}_{L}$	${\hat{\sigma }}_{U}$
Cross-prediction	0.0158	0.0182
Classical	0.0195	0.0232
PPI	0.0200	0.0240
	ACS survey analysis	
Method	${\hat{\sigma }}_{L}$	${\hat{\sigma }}_{U}$
Cross-prediction	11.2781	12.2367
Classical	14.5346	15.6106
PPI	13.1378	13.8733
	Galaxy analysis	
Method	${\hat{\sigma }}_{L}$	${\hat{\sigma }}_{U}$
Cross-prediction	0.0029	0.0029
Classical	0.0037	0.0038
PPI	0.0036	0.0037

For each problem, we take n to be the smallest labeled dataset size in the considered range. The minimum value in each column is in bold.

Finally, we provide the experimental details behind Fig. 2. We have $n_{\mathrm{all}}=3,192$ data points with gold-standard labels total. To simulate having unlabeled images, in each trial we randomly split the data into n points to serve as the labeled data, for varying $n\in \{0.1n_{\mathrm{all}},0.2n_{\mathrm{all}},0.3n_{\mathrm{all}}\}$, and treat the remaining points as unlabeled. The target of inference is the fraction of deforested areas across the locations contained in the sample. We apply cross-prediction with K=10 folds. For prediction-powered inference, we use $n_{\mathrm{tr}}=0.1n$ points for model tuning. We average the results over 100 trials.

### Estimating Relationships between Socioeconomic Covariates in Survey Data.

**Fig. 6.**
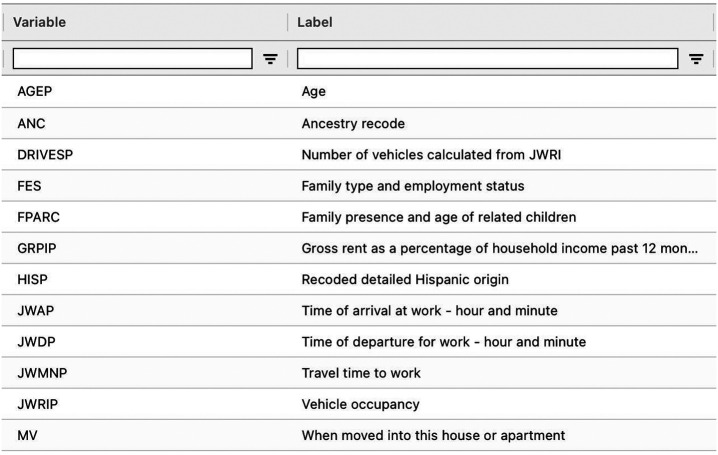
Subset of the variables available in the ACS PUMS data.

We evaluate cross-prediction on the American Community Survey (ACS) Public Use Microdata Sample (PUMS). We investigate the relationship between age, sex, and income in survey data collected in California in 2019 ($n_{\mathrm{all}}=377,575$ people total). High-quality survey data are generally difficult and time-consuming to collect. With this experiment, we hope to demonstrate how, by imputing missing information based on the available covariates, cross-prediction can achieve both powerful and valid inferences while reducing the requisite amount of survey data. See Fig. 6 for a subset of the available covariates in the ACS PUMS data.

We use the Folktables (58) interface to download the PUMS data, including income, age, sex, and 15 other demographic covariates. In each trial, we randomly sample n data points to serve as the labeled data, for varying n, and treat the remaining data points as the unlabeled data. We vary $n\in \{0.1n_{\mathrm{all}},0.2n_{\mathrm{all}},0.3n_{\mathrm{all}}\}$. The target of inference is the linear regression coefficient when regressing income on age and sex: $\theta^{*}{=}_{\theta }E\left[{\left(Y-X_{\mathrm{ols}}^{⊤}\theta \right)}^{2}\right]$, where Y is income and $X_{\mathrm{ols}}$ encodes age and sex, $X_{\mathrm{ols}}=\left(X_{\mathrm{age}},X_{\mathrm{sex}}\right)$. For the purpose of evaluating coverage, the corresponding coefficient computed on the whole dataset is taken as the ground-truth value of the estimand. To obtain the models $f^{\left(j\right)}$, we train gradient-boosted trees via XGBoost (57). Note that the predictors use all 17 covariates to predict the missing labels, even though the target of inference is only defined with respect to two covariates. We apply cross-prediction with K=5 folds. For prediction-powered inference, we use $n_{\mathrm{tr}}=0.2n$ points for model training, and we also train gradient-boosted trees. The target error level is α=0.1 and we average the results over 100 trials.

**Fig. 7.**
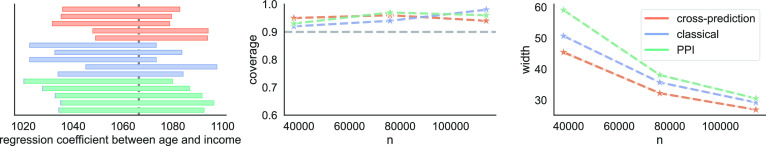
Estimating the relationship between age, sex, and income in ACS data. Intervals from five randomly chosen trials (*Left*), coverage (*Middle*), and average interval width (*Right*) of cross-prediction, classical inference, and prediction-powered inference (PPI) in a linear regression problem on ACS PUMS data. The target $\theta^{*}$ is the linear regression coefficient when regressing income on age and sex.

In Fig. 7 we plot the coverage and interval width for the three baselines, together with five example intervals. All three methods cover the true target with the desired probability. Moreover, as before, cross-prediction outperforms prediction-powered inference. In this example, the predictive power of the trained models is not high enough for prediction-powered inference to outperform the classical approach; cross-prediction, however, outperforms both. In Table 4, we report on the stability of the three methods for $n=0.1n_{\mathrm{all}}$. We observe that cross-prediction is more stable than both alternatives. We also observe that prediction-powered inference has more stable intervals than the classical approach, despite the fact that they are wider on average.

### Estimating the Prevalence of Spiral Galaxies from Galaxy Images.

**Fig. 8.**
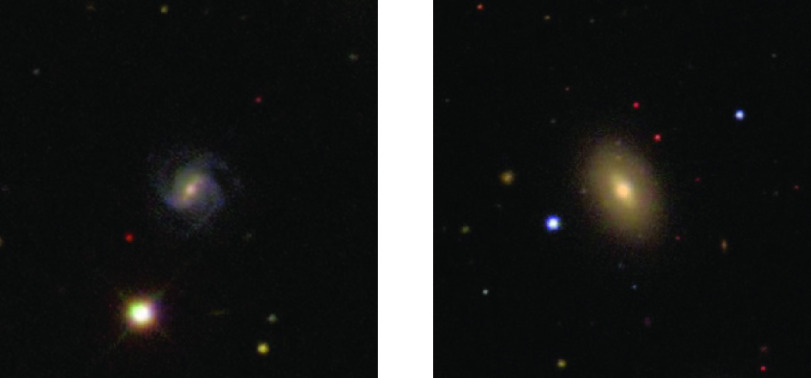
Example images of a spiral galaxy (*Left*) and a nonspiral galaxy (*Right*).

We next look at galaxy data from the Galaxy Zoo 2 dataset (59), consisting of human-annotated images of galaxies from the Sloan Digital Sky Survey (60). Of particular interest are galaxies with spiral arms, which are correlated with star formation in the discs of low-redshift galaxies, and thus contribute to the understanding of star formation. See Fig. 8 for example images of a spiral and a nonspiral galaxy. We show that, by leveraging the massive amounts of unlabeled galaxy imagery together with machine learning, cross-prediction can decrease the requisite number of human annotations for inference on galaxy demographics.

We have 167,434 annotated galaxy images. In each trial, we randomly split them up into n points to serve as the labeled data, for n∈{10,000,20,000,30,000}, and treat the remaining data points as unlabeled. The target of inference is the fraction of spiral galaxies in the dataset, equal to about 26.22%. To compute predictions, we fine-tune all layers of a pretrained ResNet50. We apply cross-prediction with K=3 folds. For prediction-powered inference, we use $n_{\mathrm{tr}}=0.1n$ points for model training. The target error rate is α=0.1 and we average the results over 100 trials.

**Fig. 9.**
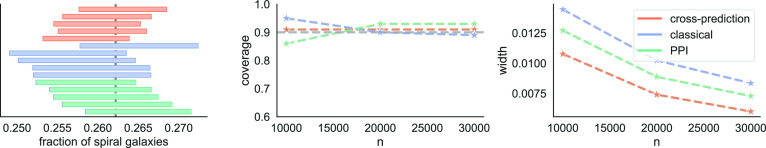
Estimating the prevalence of spiral galaxies from galaxy images. Intervals from five randomly chosen trials (*Left*), coverage (*Middle*), and average interval width (*Right*) of cross-prediction, classical inference, and prediction-powered inference (PPI) in a mean estimation problem on galaxy image data. The target $\theta^{*}$ is the fraction of spiral galaxies.

In Fig. 9 we plot the coverage and interval width of the three methods, as well as the intervals for five randomly chosen trials. Both cross-prediction and prediction-powered inference yield smaller intervals than the classical approach. Moreover, cross-prediction dominates prediction-powered inference. We observe satisfactory coverage for all three procedures. In Table 4 we evaluate the stability of the procedures for n=10,000. Cross-prediction is significantly more stable than classical inference and prediction-powered inference. The latter two achieve a similar degree of stability.

## Evaluating Heuristics

In Fig. 2, we saw that cross-prediction gave tighter CIs than the baseline approaches for the problem of deforestation analysis. In this section, we test two heuristic ways of reducing the variance of the classical approach and prediction-powered inference and compare the heuristics to cross-prediction.

The first heuristic removes the debiasing from the cross-prediction estimator and simply averages the predictions on the large unlabeled dataset:

[10] $$\begin{array}{c} {\hat{\theta }}_{\mathrm{nodebias}}=\frac{1}{\mathrm{KN}}\sum_{j=1}^{K}\sum_{i=1}^{N}f^{\left(j\right)}\left({\tilde{X}}_{i}\right). \end{array}$$

This is akin to computing the classical estimator while pretending that the predicted labels are the ground truth. The second heuristic trains a model on all the labeled data, $f^{\mathrm{all}}=A_{\mathrm{train}}\left({\left\{\left(X_{i},Y_{i}\right)\right\}}_{i=1}^{n}\right)$, and computes

$$\begin{array}{c} {\hat{\theta }}_{\mathrm{nofolds}}=\frac{1}{N}\sum_{i=1}^{N}f^{\mathrm{all}}\left({\tilde{X}}_{i}\right)-\frac{1}{n}\sum_{i=1}^{n}\left(f^{\mathrm{all}}\left(X_{i}\right)-Y_{i}\right). \end{array}$$

This estimator is akin to the prediction-powered estimator if we treated $f^{\mathrm{all}}$ as fixed and independent of the labeled dataset.

For both heuristics, we form confidence intervals based on the usual central limit theorem that assumes i.i.d. sampling. For the first heuristic this is done conditional on the trained models $f^{\left(j\right)}$, since the terms ${\left(\frac{1}{K}\sum_{j=1}^{K}f^{\left(j\right)}\left({\tilde{X}}_{i}\right)\right)}_{i\in [N]}$ are indeed conditionally independent given $f^{\left(1\right)},\cdots ,f^{\left(K\right)}$. Since the second heuristic proceeds under the assumption that $f^{\mathrm{all}}$ can be seen as being independent of the labeled data, we apply the central limit theorem to the two sums separately, as if $f^{\mathrm{all}}$ were fixed.

**Fig. 10.**
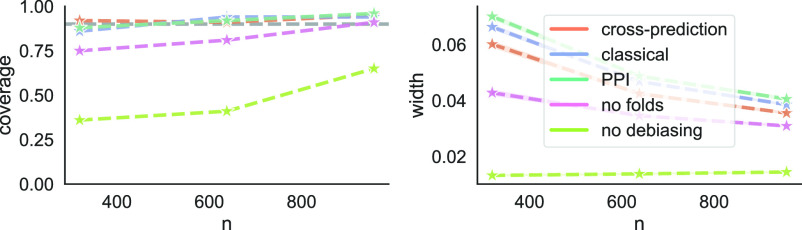
Estimating the deforestation rate in the Amazon from satellite imagery (revisited). Coverage and average interval width of cross-prediction, classical inference, and prediction-powered inference (PPI), as well as two heuristics related to cross-fitting: one that removes the debiasing and one that trains on all labeled data instead of forming folds. The experimental setup is the same as in Fig. 2.

We see in Fig. 10 that removing the debiasing is detrimental to coverage; removing the folds has a more moderate effect that vanishes with n, but it is nevertheless significant. Cross-prediction yields wider intervals than both heuristics, and by doing so it maintains correct coverage.

## Supplementary Material

Appendix 01 (PDF)

## Data Availability

Code and data for reproducing the experiments have been deposited in cross-ppi GitHub repository (56).

## Footnotes

^*^By removing at most K−1 data points, the size of the labeled dataset can be made divisible by K. Since in our applications K will typically be equal to 10, this truncation has a negligible effect.
