# Supplementary material for: Cross-prediction-powered inference
Source: Proc Natl Acad Sci U S A. 2024 Apr 3;121(15):e2322083121. doi: 10.1073/pnas.2322083121 (PMC11009639; doi:10.1073/pnas.2322083121)
Supplement: Supplementary file 1 — Appendix 01 (PDF) [file pnas.2322083121.sapp.pdf]

Supporting Information for

Cross-prediction-powered inference

Tijana Zrnic, Emmanuel J. Candès

Emmanuel J. Candès.
E-mail: candes@stanford.edu

This PDF file includes:

Supporting text
SI References

Supporting Information Text

Proof of Theorem 1 (CLT for mean estimation)

The proof builds on two key technical lemmas, which leverage the notion of stability in Assumption 1 to show that we can "replace" the models $f^{(j)}$ in the definition of the cross-prediction estimator with the "average" model $\bar{f}$. Since $\bar{f}$ is a nonrandom model, we can proceed with a standard CLT analysis of the two terms comprising the estimator.

We begin by stating and proving the technical lemmas, which are inspired by the analysis of cross-validation due to Bayle et al. (1). To simplify notation, we use $\mathbb{E}_X$ and Var_X to denote the expectation and variance conditional on everything but X.

Lemma 1. *Suppose that the predictions are stable (Ass. 1). Denote*

$$\tilde{F}_j = \frac{1}{\sqrt{N}} \sum_{i=1}^{N} \left(f^{(j)}(\tilde{X}_i) - \mathbb{E}_X[f^{(j)}(X)] - (\bar{f}(\tilde{X}_i) - \mathbb{E}[\bar{f}(X)]) \right). \quad [1]$$

Then,

$$\frac{1}{K} \sum_{j=1}^{K} \tilde{F}_j \xrightarrow{p} 0.$$

Proof. Let $\psi(x) = \min(1, x)$. We will use the fact that $\frac{1}{K}\sum_{j=1}^K \tilde{F}_j \xrightarrow{p} 0$ if and only if $\mathbb{E}[\psi(|\frac{1}{K}\sum_{j=1}^K \tilde{F}_j|)] \to 0$, as stated in Fact 1 below. See, for example, Bayle et al. (1) for a proof of the fact.

Fact 1. *Let X_n be a sequence of random variables. Then, $X_n \xrightarrow{p} 0$ if and only if* $\mathbb{E}[\min(1, |X_n|)] \to 0$.

Note that $\psi(x)$ is nondecreasing and satisfies $\psi\left(\sum_{j=1}^K x_j\right) \leq \sum_{j=1}^K \psi(x_j)$ for non-negative x_j; this yields

$$\mathbb{E}\left[\psi\left(\left|\frac{1}{K}\sum_{j=1}^K \tilde{F}_j\right|\right)\right] \leq \mathbb{E}\left[\psi\left(\frac{1}{K}\sum_{j=1}^K |\tilde{F}_j|\right)\right] \leq \sum_{j=1}^K \mathbb{E}\left[\psi\left(\frac{1}{K}|\tilde{F}_j|\right)\right].$$

Notice that $\psi(x)$ is also concave. Therefore, by Jensen's inequality, we have

$$\begin{aligned}
\sum_{j=1}^K \mathbb{E}\left[\psi\left(\frac{1}{K}|\tilde{F}_j|\right)\right] &\leq \sum_{j=1}^K \mathbb{E}\left[\psi\left(\frac{1}{K}\mathbb{E}\left[|\tilde{F}_j| \middle| f^{(j)}\right]\right)\right] \\
&\leq \sum_{j=1}^K \mathbb{E}\left[\psi\left(\frac{1}{K}\sqrt{\mathbb{E}[\tilde{F}_j^2 | f^{(j)}]}\right)\right] \\
&= \sum_{j=1}^K \mathbb{E}\left[\psi\left(\frac{1}{K}\sqrt{\mathrm{Var}(\tilde{F}_j | f^{(j)})}\right)\right] \\
&= \sum_{j=1}^K \mathbb{E}\left[\psi\left(\frac{1}{K}\sqrt{\mathrm{Var}_X\left(f^{(j)}(X) - \bar{f}(X)\right)}\right)\right] \\
&= \mathbb{E}\left[\min\left(K, \sqrt{\mathrm{Var}_X\left(f^{(1)}(X) - \bar{f}(X)\right)}\right)\right].
\end{aligned}$$

Invoking the stability condition shows that the right-hand side converges to zero. Note that, technically, the stability condition is stronger than what is needed for the expression above to converge to zero. In particular, stability ensures that $\mathbb{E}\left[\sqrt{K \cdot \mathrm{Var}_X\left(f^{(1)}(X) - \bar{f}(X)\right)}\right] \to 0$, while for the expression above to vanish it would suffice to ensure $\mathbb{E}\left[\sqrt{\mathrm{Var}_X\left(f^{(1)}(X) - \bar{f}(X)\right)}\right] \to 0$. The stronger condition will be used in the next technical lemma, which handles the second term in the cross-prediction estimator.

Putting everything together, we get that $\frac{1}{K}\sum_{j=1}^K \tilde{F}_j \xrightarrow{p} 0$, as desired. □

Lemma 2. *Suppose that the predictions are stable (Ass. 1). Denote*

$$F_j = \frac{1}{\sqrt{n}} \sum_{i \in I_j} \left(f^{(j)}(X_i) - \mathbb{E}_X[f^{(j)}(X)] - (\bar{f}(X_i) - \mathbb{E}[\bar{f}(X)]) \right). \quad [2]$$

Then,

$$\sum_{j=1}^{K} F_j \xrightarrow{p} 0.$$

Proof. The proof follows a similar principle as the proof of Lemma 1. As before, we let $\psi(x) = \min(1, x)$ and use the fact $\sum_{j=1}^K F_j \xrightarrow{p} 0$ if and only if $\mathbb{E}[\psi(|\sum_{j=1}^K F_j|)] \to 0$ (see Fact 1). We use the fact that $\psi\left(\sum_{j=1}^K x_j\right) \le \sum_{j=1}^K \psi(x_j)$ for non-negative x_j; this yields

$$\mathbb{E}\left[\psi\left(\left|\sum_{j=1}^K F_j\right|\right)\right] \le \mathbb{E}\left[\psi\left(\sum_{j=1}^K |F_j|\right)\right] \le \sum_{j=1}^K \mathbb{E}\left[\psi\left(|F_j|\right)\right].$$

Next, by Jensen's inequality, we have

$$\begin{aligned}
\sum_{j=1}^K \mathbb{E}\left[\psi\left(|F_j|\right)\right] &\le \sum_{j=1}^K \mathbb{E}\left[\psi\left(\mathbb{E}[|F_j| \mid f^{(j)}]\right)\right] \\
&\le \sum_{j=1}^K \mathbb{E}\left[\psi\left(\sqrt{\mathbb{E}[F_j^2 | f^{(j)}]}\right)\right] \\
&= \sum_{j=1}^K \mathbb{E}\left[\psi\left(\sqrt{\mathrm{Var}(F_j | f^{(j)})}\right)\right] \\
&= \sum_{j=1}^K \mathbb{E}\left[\psi\left(\sqrt{\frac{|I_j|}{n}\mathrm{Var}_X\left(f^{(j)}(X) - \bar{f}(X)\right)}\right)\right] \\
&= \mathbb{E}\left[\min\left(K, \sqrt{K \cdot \mathrm{Var}_X\left(f^{(1)}(X) - \bar{f}(X)\right)}\right)\right].
\end{aligned}$$

Invoking the stability condition shows that the right-hand side converges to zero. Hence, $\sum_{j=1}^K F_j \xrightarrow{p} 0$. □

With Lemma 1 and Lemma 2 in hand, we can now prove Theorem 1. As alluded to earlier, the idea is to use Lemma 1 and Lemma 2 to replace the models $f^{(j)}$ in the definition of the cross-prediction estimator.

Writing $\theta^* = \frac{1}{K}\sum_{j=1}^K (\mathbb{E}[f^{(j)}(X)] - \mathbb{E}[f^{(j)}(X) - Y])$, we have

$$\begin{aligned}
&\frac{\sqrt{n}}{\sqrt{\frac{n}{N}\bar{\sigma}^2 + \bar{\sigma}_\Delta^2}}\left(\hat{\theta}^+ - \theta^*\right) \\
&= \frac{\sqrt{n}}{\sqrt{\frac{n}{N}\bar{\sigma}^2 + \bar{\sigma}_\Delta^2}}\left(\frac{1}{KN}\sum_{j=1}^K\sum_{i=1}^N (f^{(j)}(\tilde{X}_i) - \mathbb{E}_X[f^{(j)}(X)]) - \frac{1}{n}\sum_{j=1}^K\sum_{i\in I_j}((f^{(j)}(X_i) - Y_i) - \mathbb{E}_{X,Y}[f^{(j)}(X) - Y])\right) \\
&= \frac{\sqrt{n}}{\sqrt{\frac{n}{N}\bar{\sigma}^2 + \bar{\sigma}_\Delta^2}}(T_1 - T_2), \qquad [3]
\end{aligned}$$

where we define

$$T_1 = \frac{1}{KN}\sum_{j=1}^K\sum_{i=1}^N (f^{(j)}(\tilde{X}_i) - \mathbb{E}_X[f^{(j)}(X)]); \quad T_2 = \frac{1}{n}\sum_{j=1}^K\sum_{i\in I_j}((f^{(j)}(X_i) - Y_i) - \mathbb{E}_{X,Y}[f^{(j)}(X) - Y]).$$

Focusing on T_1, we have

$$\sqrt{N}T_1 = \frac{1}{K}\sum_{j=1}^K \tilde{F}_j + \frac{1}{\sqrt{N}}\sum_{i=1}^N (\bar{f}(\tilde{X}_i) - \mathbb{E}[\bar{f}(X)]),$$

for $\tilde{F}_j$ defined in Eq. (1). Invoking Lemma 1, we thus get $\sqrt{N}T_1 = \frac{1}{\sqrt{N}}\sum_{i=1}^N (\bar{f}(\tilde{X}_i) - \mathbb{E}[\bar{f}(X)]) + o_P(1)$.

By an analogous argument, we have

$$\begin{aligned}
\sqrt{n}T_2 &= \sum_{j=1}^K F_j + \frac{1}{\sqrt{n}}\sum_{j=1}^K\sum_{i\in I_j}\left((\bar{f}(X_i) - Y_i) - \mathbb{E}_{X,Y}[\bar{f}(X) - Y]\right) \\
&= \frac{1}{\sqrt{n}}\sum_{i=1}^n \left((\bar{f}(X_i) - Y_i) - \mathbb{E}_{X,Y}[\bar{f}(X) - Y]\right) + o_P(1),
\end{aligned}$$

for F_j defined in Eq. (2). Going back to Eq. (3) and denoting by $r, \bar{\sigma}^2_{\mathrm{lim}}, \bar{\sigma}^2_{\Delta,\mathrm{lim}}$ the limits of $\frac{n}{N}, \bar{\sigma}^2, \bar{\sigma}^2_\Delta$, respectively, we get

$$\begin{aligned}
&\frac{\sqrt{n}}{\sqrt{\frac{n}{N}\bar{\sigma}^2 + \bar{\sigma}^2_\Delta}}\left(\hat{\theta}^+ - \theta^*\right) \\
&= \frac{\sqrt{\frac{n}{N}}}{\sqrt{\frac{n}{N}\bar{\sigma}^2 + \bar{\sigma}^2_\Delta}}\sqrt{N}T_1 - \frac{1}{\sqrt{\frac{n}{N}\bar{\sigma}^2 + \bar{\sigma}^2_\Delta}}\sqrt{n}T_2 \\
&= \frac{\sqrt{\frac{n}{N}}}{\sqrt{\frac{n}{N}\bar{\sigma}^2 + \bar{\sigma}^2_\Delta}}\frac{1}{\sqrt{N}}\sum_{i=1}^{N}(\bar{f}(\tilde{X}_i) - \mathbb{E}[\bar{f}(X)]) - \frac{1}{\sqrt{\frac{n}{N}\bar{\sigma}^2 + \bar{\sigma}^2_\Delta}}\frac{1}{\sqrt{n}}\sum_{i=1}^{n}\left((\bar{f}(X_i) - Y_i) - \mathbb{E}_{X,Y}[\bar{f}(X) - Y]\right) + o_P(1).
\end{aligned}$$

By the Lindeberg central limit theorem, the first term converges in distribution to $\mathcal{N}\left(0, \frac{r\bar{\sigma}^2_{\mathrm{lim}}}{r\bar{\sigma}^2_{\mathrm{lim}} + \bar{\sigma}^2_{\Delta,\mathrm{lim}}}\right)$, and the second term converges in distribution to $\mathcal{N}\left(0, \frac{\bar{\sigma}^2_{\Delta,\mathrm{lim}}}{r\bar{\sigma}^2_{\mathrm{lim}} + \bar{\sigma}^2_{\Delta,\mathrm{lim}}}\right)$. Moreover, since the two terms are independent, we finally have

$$\frac{\sqrt{n}}{\sqrt{\frac{n}{N}\bar{\sigma}^2 + \bar{\sigma}^2_\Delta}}\left(\hat{\theta}^+ - \theta^*\right) \xrightarrow{d} \mathcal{N}(0,1).$$

Proof of Theorem 2 (CLT for general M-estimation)

The proof follows a similar template as the proof of Theorem 1. We begin with two technical lemmas that allow swapping the models $f^{(j)}$ in the gradient of the cross-prediction loss, $\nabla L^+(\theta)$, with the "average" model $\bar{f}$.

Lemma 3. *Suppose that the predictions are stable (Ass. 2). Denote*

$$\tilde{L}_j = \frac{1}{\sqrt{N}}\sum_{i=1}^{N}\left(\nabla\tilde{\ell}^{f^{(j)}}_{\theta,i} - \mathbb{E}[\nabla\ell^{f^{(j)}}_\theta] - (\nabla\tilde{\ell}^{\bar{f}}_{\theta,i} - \mathbb{E}[\nabla\ell^{\bar{f}}_\theta])\right). \qquad [4]$$

Then,

$$\frac{1}{K}\sum_{j=1}^{K}\tilde{L}_j \xrightarrow{p} 0.$$

Lemma 4. *Suppose that the predictions are stable (Ass. 2). Denote*

$$L_j = \frac{1}{\sqrt{n}}\sum_{i\in I_j}\left(\nabla\ell^{f^{(j)}}_{\theta,i} - \mathbb{E}[\nabla\ell^{f^{(j)}}_\theta] - (\nabla\ell^{\bar{f}}_{\theta,i} - \mathbb{E}[\nabla\ell^{\bar{f}}_\theta])\right). \qquad [5]$$

Then,

$$\sum_{j=1}^{K}L_j \xrightarrow{p} 0.$$

Lemma 3 and Lemma 4 are proved completely analogously to Lemma 1 and Lemma 2; we apply the same argument as before entry-wise.

Now we put the lemmas together to prove the central limit theorems. We first analyze the asymptotic normality of $\nabla L^+(\theta)$. The asymptotic normality of $\hat{\theta}^+$ relies on a similar application of the lemmas.

Asymptotic normality of $\nabla L^+(\theta)$. We can write

$$\begin{aligned}
&\sqrt{n}\left(\frac{n}{N}\bar{\Sigma}_\theta + \bar{\Sigma}_{\Delta,\theta}\right)^{-1/2}(\nabla L^+(\theta) - \nabla L(\theta)) \\
&= \sqrt{n}\left(\frac{n}{N}\bar{\Sigma}_\theta + \bar{\Sigma}_{\Delta,\theta}\right)^{-1/2}\left(\frac{1}{KN}\sum_{j=1}^{K}\sum_{i=1}^{N}\nabla\tilde{\ell}^{f^{(j)}}_{\theta,i} - \frac{1}{n}\sum_{j=1}^{K}\sum_{i\in I_j}(\nabla\ell^{f^{(j)}}_{\theta,i} - \nabla\ell_{\theta,i}) - \nabla L(\theta)\right) \\
&= \sqrt{n}\left(\frac{n}{N}\bar{\Sigma}_\theta + \bar{\Sigma}_{\Delta,\theta}\right)^{-1/2}\left(\frac{1}{KN}\sum_{j=1}^{K}\sum_{i=1}^{N}(\nabla\tilde{\ell}^{f^{(j)}}_{\theta,i} - \mathbb{E}[\nabla\ell^{f^{(j)}}_\theta]) - \frac{1}{n}\sum_{j=1}^{K}\sum_{i\in I_j}((\nabla\ell^{f^{(j)}}_{\theta,i} - \nabla\ell_{\theta,i}) - \mathbb{E}[\nabla\ell^{f^{(j)}}_\theta - \nabla\ell_\theta])\right) \\
&= \sqrt{n}\left(\frac{n}{N}\bar{\Sigma}_\theta + \bar{\Sigma}_{\Delta,\theta}\right)^{-1/2}(T_1 - T_2),
\end{aligned} \qquad [6]$$

where we define

$$T_1 = \frac{1}{KN}\sum_{j=1}^{K}\sum_{i=1}^{N}(\nabla\tilde{\ell}_{\theta,i}^{f^{(j)}} - \mathbb{E}[\nabla\ell_{\theta}^{f^{(j)}}]); \quad T_2 = \frac{1}{n}\sum_{j=1}^{K}\sum_{i\in I_j}((\nabla\ell_{\theta,i}^{f^{(j)}} - \nabla\ell_{\theta,i}) - \mathbb{E}[\nabla\ell_{\theta}^{f^{(j)}} - \nabla\ell_{\theta}]).$$

We apply Lemma 3 and Lemma 4 to T_1 and T_2, respectively. In particular, we can write

$$\sqrt{N}T_1 = \frac{1}{K}\sum_{j=1}^{K}\tilde{L}_j + \frac{1}{\sqrt{N}}\sum_{i=1}^{N}(\nabla\tilde{\ell}_{\theta,i}^{\bar{f}} - \mathbb{E}[\nabla\ell_{\theta}^{\bar{f}}]),$$

where $\tilde{L}_j$ is given in Eq. (4). Invoking Lemma 3, we thus have $\sqrt{N}T_1 = \frac{1}{\sqrt{N}}\sum_{i=1}^{N}(\nabla\tilde{\ell}_{\theta,i}^{\bar{f}} - \mathbb{E}[\nabla\ell_{\theta}^{\bar{f}}]) + o_P(1)$.

By an analogous argument, for L_j defined in Eq. (5), we have

$$\begin{aligned}\sqrt{n}T_2 &= \sum_{j=1}^{K}L_j + \frac{1}{\sqrt{n}}\sum_{j=1}^{K}\sum_{i\in I_j}\left((\nabla\ell_{\theta,i}^{\bar{f}} - \nabla\ell_{\theta,i}) - \mathbb{E}[\nabla\ell_{\theta}^{\bar{f}} - \nabla\ell_{\theta}]\right)\\ &= \frac{1}{\sqrt{n}}\sum_{i=1}^{n}\left((\nabla\ell_{\theta,i}^{\bar{f}} - \nabla\ell_{\theta,i}) - \mathbb{E}[\nabla\ell_{\theta}^{\bar{f}} - \nabla\ell_{\theta}]\right) + o_P(1).\end{aligned}$$

Going back to Eq. (6) and denoting by $r, \bar{\Sigma}_{\theta,\mathrm{lim}}, \bar{\Sigma}_{\Delta,\theta,\mathrm{lim}}$ the limits of $\frac{n}{N}, \bar{\Sigma}_{\theta}, \bar{\Sigma}_{\Delta,\theta}$, respectively, by the Lindeberg central limit theorem we get

$$\begin{aligned}\sqrt{n}\left(\frac{n}{N}\bar{\Sigma}_{\theta} + \bar{\Sigma}_{\Delta,\theta}\right)^{-1/2}\left(\nabla L^{+}(\theta) - \nabla L(\theta)\right) &= \left(\frac{n}{N}\bar{\Sigma}_{\theta} + \bar{\Sigma}_{\Delta,\theta}\right)^{-1/2}\left(\frac{\sqrt{n}}{\sqrt{N}}\sqrt{N}T_1 - \sqrt{n}T_2\right)\\ &= \left(\frac{n}{N}\bar{\Sigma}_{\theta} + \bar{\Sigma}_{\Delta,\theta}\right)^{-1/2}\sqrt{\frac{n}{N}}\frac{1}{\sqrt{N}}\sum_{i=1}^{N}(\nabla\tilde{\ell}_{\theta,i}^{\bar{f}} - \mathbb{E}[\nabla\ell_{\theta}^{\bar{f}}])\\ &\quad - \left(\frac{n}{N}\bar{\Sigma}_{\theta} + \bar{\Sigma}_{\Delta,\theta}\right)^{-1/2}\frac{1}{\sqrt{n}}\sum_{i=1}^{n}\left((\nabla\ell_{\theta,i}^{\bar{f}} - \nabla\ell_{\theta,i}) - \mathbb{E}[\nabla\ell_{\theta}^{\bar{f}} - \nabla\ell_{\theta}]\right)\end{aligned}$$

The first term above converges in distribution to

$$\mathcal{N}\left(0, r\cdot(r\bar{\Sigma}_{\theta,\mathrm{lim}} + \bar{\Sigma}_{\Delta,\theta,\mathrm{lim}})^{-1/2}\bar{\Sigma}_{\theta,\mathrm{lim}}(r\bar{\Sigma}_{\theta,\mathrm{lim}} + \bar{\Sigma}_{\Delta,\theta,\mathrm{lim}})^{-1/2}\right),$$

and the second term converges in distribution to

$$\mathcal{N}\left(0, (r\bar{\Sigma}_{\theta,\mathrm{lim}} + \bar{\Sigma}_{\Delta,\theta,\mathrm{lim}})^{-1/2}\bar{\Sigma}_{\Delta,\theta,\mathrm{lim}}(r\bar{\Sigma}_{\theta,\mathrm{lim}} + \bar{\Sigma}_{\Delta,\theta,\mathrm{lim}})^{-1/2}\right).$$

Since the two terms are independent, we can add up their limiting covariance matrices and get

$$\sqrt{n}\left(\frac{n}{N}\bar{\Sigma}_{\theta} + \bar{\Sigma}_{\Delta,\theta}\right)^{-1/2}\left(\nabla L^{+}(\theta) - \nabla L(\theta)\right) \xrightarrow{d} \mathcal{N}(0, I),$$

as desired.

Asymptotic normality of $\hat{\theta}^{+}$. We follow an argument similar to the classical proof of asymptotic normality of M-estimators (see Theorem 5.23 in (2)). Given a function g, we use the shorthand notation

$$\begin{aligned}\mathbb{E}_n g &:= \frac{1}{n}\sum_{i=1}^{n}g(X_i, Y_i), \quad \mathbb{G}_n g = \sqrt{n}\left(\mathbb{E}_n g - \mathbb{E}[g(X,Y)]\right);\\ \widetilde{\mathbb{E}}_N^{f^+} g &:= \frac{1}{NK}\sum_{i=1}^{N}\sum_{j=1}^{K}g(\widetilde{X}_i, f^{(j)}(\widetilde{X}_i)), \quad \widetilde{\mathbb{G}}_N^{f^+} g := \sqrt{N}\left(\widetilde{\mathbb{E}}_N^{f^+} g - \frac{1}{K}\sum_{j=1}^{K}\mathbb{E}_X[g(X, f^{(j)}(X))]\right);\\ \widetilde{\mathbb{E}}_N^{\bar{f}} g &:= \frac{1}{N}\sum_{i=1}^{N}g(\widetilde{X}_i, \bar{f}(\widetilde{X}_i)), \quad \widetilde{\mathbb{G}}_N^{\bar{f}} g := \sqrt{N}\left(\widetilde{\mathbb{E}}_N^{\bar{f}} g - \mathbb{E}[g(X, \bar{f}(X))]\right);\\ \mathbb{E}_n^{f^+} g &:= \frac{1}{n}\sum_{j=1}^{K}\sum_{i\in I_j}g(X_i, f^{(j)}(X_i)), \quad \mathbb{G}_n^{f^+} g := \sqrt{n}\left(\mathbb{E}_n^{f^+} g - \frac{1}{K}\sum_{j=1}^{K}\mathbb{E}_X[g(X, f^{(j)}(X))]\right);\\ \mathbb{E}_n^{\bar{f}} g &:= \frac{1}{n}\sum_{i=1}^{n}g(X_i, \bar{f}(X_i)), \quad \mathbb{G}_n^{\bar{f}} g := \sqrt{n}(\mathbb{E}_n^{\bar{f}} g - \mathbb{E}[g(X, \bar{f}(X))]).\end{aligned}$$

The differentiability and local Lipschitzness of the loss at θ^* imply that for every (possibly random) sequence $h_n = O_P(1)$, we have

$$\mathbb{G}_n^{f^+}\left[\sqrt{n}\left(\ell_{\theta^*+\frac{h_n}{\sqrt{n}}} - \ell_{\theta^*}\right)\right] = \mathbb{G}_n^{f^+}\left[h_n^\top \nabla \ell_{\theta^*}\right] + o_P(1).$$

By essentially the same argument as in the proof of the asymptotic normality of $\nabla L^+(\theta)$, we can substitute the average over models $f^{(j)}$ for $\bar{f}$ via Lemma 4, thus getting

$$\mathbb{G}_n^{f^+}\left[h_n^\top \nabla \ell_{\theta^*}\right] = \mathbb{G}_n^{\bar{f}}\left[h_n^\top \nabla \ell_{\theta^*}\right] + o_P(1).$$

Analogously, we have $\mathbb{G}_n[\sqrt{n}(\ell_{\theta^*+h_n/\sqrt{n}} - \ell_{\theta^*})] = \mathbb{G}_n[h_n^\top \nabla \ell_{\theta^*}] + o_P(1)$, and, by using Lemma 3,

$$\tilde{\mathbb{G}}_N^{f^+}[\sqrt{n}(\ell_{\theta^*+h_n/\sqrt{n}} - \ell_{\theta^*})] = \tilde{\mathbb{G}}_N^{\bar{f}}[h_n^\top \nabla \ell_{\theta^*}] + o_P(1).$$

Next, denoting $H_{\theta^*}^{f^+} = \frac{1}{K}\sum_{j=1}^K \nabla^2 \mathbb{E}_X[\ell_{\theta^*}(X, f^{(j)}(X))]$, we apply a second-order Taylor expansion to get

$$\begin{aligned}
n\mathbb{E}_n\left(\ell_{\theta^*+\frac{h_n}{\sqrt{n}}} - \ell_{\theta^*}\right) &= \frac{1}{2}h_n^\top H_{\theta^*} h_n + h_n^\top \mathbb{G}_n \nabla \ell_{\theta^*} + o_P(1);\\
n\widetilde{\mathbb{E}}_N^{f^+}\left(\ell_{\theta^*+\frac{h_n}{\sqrt{n}}} - \ell_{\theta^*}\right) &= \frac{1}{2}h_n^\top H_{\theta^*}^{f^+} h_n + \sqrt{\frac{n}{N}} h_n^\top \widetilde{\mathbb{G}}_N^{\bar{f}} \nabla \ell_{\theta^*} + o_P(1);\\
-n\mathbb{E}_n^{f^+}\left(\ell_{\theta^*+\frac{h_n}{\sqrt{n}}} - \ell_{\theta^*}\right) &= -\frac{1}{2}h_n^\top H_{\theta^*}^{f^+} h_n - h_n^\top \mathbb{G}_n^{\bar{f}} \nabla \ell_{\theta^*} + o_P(1).
\end{aligned}$$

Notice that we have $L^+(\theta) = \tilde{\mathbb{E}}_N^{f^+}\ell_\theta + \mathbb{E}_n \ell_\theta - \mathbb{E}_n^{f^+}\ell_\theta$. Thus, if we add up the three equations above, we get the following:

$$n\left(L^+\left(\theta^* + \frac{h_n}{\sqrt{n}}\right) - L^+(\theta^*)\right) = \frac{1}{2}h_n^\top H_{\theta^*} h_n + h_n^\top\left(\mathbb{G}_n \nabla \ell_{\theta^*} + \sqrt{\frac{n}{N}}\tilde{\mathbb{G}}_N^{\bar{f}} \nabla \ell_{\theta^*} - \mathbb{G}_n^{\bar{f}} \nabla \ell_{\theta^*}\right) + o_P(1).$$

We now evaluate this expression for two values of h_n, $h_n^* = \sqrt{n}(\hat{\theta}^+ - \theta^*)$ and $h_n' = -H_{\theta^*}^{-1}\left(\mathbb{G}_n \nabla \ell_{\theta^*} + \sqrt{\frac{n}{N}}\tilde{\mathbb{G}}_N^{\bar{f}} \nabla \ell_{\theta^*} - \mathbb{G}_n^{\bar{f}} \nabla \ell_{\theta^*}\right)$. This gives

$$\begin{aligned}
n\left(L^+(\hat{\theta}^+) - L^+(\theta^*)\right) &= \frac{1}{2}(h_n^*)^\top H_{\theta^*} h_n^* + (h_n^*)^\top\left(\mathbb{G}_n \nabla \ell_{\theta^*} + \sqrt{\frac{n}{N}}\tilde{\mathbb{G}}_N^{\bar{f}} \nabla \ell_{\theta^*} - \mathbb{G}_n^{\bar{f}} \nabla \ell_{\theta^*}\right) + o_P(1);\\
n\left(L^+(\theta^* - h_n'/\sqrt{n}) - L^+(\theta^*)\right) &= -\frac{1}{2}(h_n')^\top H_{\theta^*} h_n' + o_P(1);
\end{aligned}$$

By the definition of $\hat{\theta}^+$, the left-hand side of the first equation is smaller than the left-hand side of the second equation, hence the same relation is true for the right-hand sides. Taking the difference of the right-hand sides and completing the square gives

$$\frac{1}{2}(h_n^* - h_n')^\top H_{\theta^*}(h_n^* - h_n') + o_P(1) \leq 0.$$

Since the Hessian is positive definite, we must have $h_n^* = h_n' + o_P(1)$, that is:

$$\sqrt{n}(\hat{\theta}^+ - \theta^*) = -H_{\theta^*}^{-1}\left(\mathbb{G}_n \nabla \ell_{\theta^*} + \sqrt{\frac{n}{N}}\tilde{\mathbb{G}}_N^{\bar{f}} \nabla \ell_{\theta^*} - \mathbb{G}_n^{\bar{f}} \nabla \ell_{\theta^*}\right) + o_P(1).$$

The final statement follows by a standard application of the central limit theorem to the second term. In particular, letting $r = \lim \frac{n}{N}$, we have:

$$\begin{aligned}
&\mathbb{G}_n \nabla \ell_{\theta^*} + \sqrt{\frac{n}{N}}\tilde{\mathbb{G}}_N^{\bar{f}} \nabla \ell_{\theta^*} - \mathbb{G}_n^{\bar{f}} \nabla \ell_{\theta^*}\\
&= \frac{1}{\sqrt{n}}\sum_{i=1}^n \left(\nabla \ell_{\theta^*,i} - \nabla \ell_{\theta^*,i}^{\bar{f}} - \mathbb{E}[\nabla \ell_{\theta^*,i} - \nabla \ell_{\theta^*,i}^{\bar{f}}]\right) + \sqrt{\frac{n}{N}}\frac{1}{\sqrt{N}}\sum_{i=1}^N \left(\nabla \ell_{\theta^*,i}^{\bar{f}} - \mathbb{E}[\nabla \ell_{\theta^*}^{\bar{f}}]\right)\\
&\xrightarrow{d} \mathcal{N}\left(0, \mathrm{Var}(\nabla \ell_{\theta^*} - \nabla \ell_{\theta^*}^{\bar{f}}) + r\mathrm{Var}(\nabla \ell_{\theta^*}^{\bar{f}})\right).
\end{aligned}$$

Therefore, $-H_{\theta^*}^{-1}\left(\mathbb{G}_n \nabla \ell_{\theta^*} + \sqrt{\frac{n}{N}}\tilde{\mathbb{G}}_N^{\bar{f}} \nabla \ell_{\theta^*} - \mathbb{G}_n^{\bar{f}} \nabla \ell_{\theta^*}\right)$ converges to $\mathcal{N}(0, \bar{\Sigma})$, where

$$\bar{\Sigma} = H_{\theta^*}^{-1}\left(\bar{\Sigma}_{\Delta,\theta^*} + r\bar{\Sigma}_{\theta^*}\right)H_{\theta^*}^{-1}.$$

References

1. P Bayle, A Bayle, L Janson, L Mackey, Cross-validation confidence intervals for test error. *Adv. Neural Inf. Process. Syst.* **33**, 16339–16350 (2020).
2. AW van der Vaart, *Asymptotic Statistics*, Cambridge Series in Statistical and Probabilistic Mathematics. (Cambridge University Press), (1998).
